# Biological and phylogenetic characteristics of West African lineages of West Nile virus

**DOI:** 10.1371/journal.pntd.0006078

**Published:** 2017-11-08

**Authors:** Gamou Fall, Nicholas Di Paola, Martin Faye, Moussa Dia, Caio César de Melo Freire, Cheikh Loucoubar, Paolo Marinho de Andrade Zanotto, Ousmane Faye, Amadou Alpha Sall

**Affiliations:** 1 Pôle de Virologie, Unité des Arbovirus et virus des fièvres hémorragiques, Institut Pasteur de Dakar, Dakar, Sénégal; 2 Laboratory of Molecular Evolution and Bioinformatics, Department of Microbiology, Biomedical Sciences Institute, University of Sao Paulo, Sao Paulo, Brazil; 3 Department of Genetics and Evolution, Federal University of Sao Carlos, Sao Carlos, Brazil; 4 Groupe à 4 ans de Biostatistiques, Bioinformatique et modélisation, Institut Pasteur de Dakar, Dakar, Sénégal; University of Texas Medical Branch, UNITED STATES

## Abstract

The West Nile virus (WNV), isolated in 1937, is an arbovirus (arthropod-borne virus) that infects thousands of people each year. Despite its burden on global health, little is known about the virus’ biological and evolutionary dynamics. As several lineages are endemic in West Africa, we obtained the complete polyprotein sequence from three isolates from the early 1990s, each representing a different lineage. We then investigated differences in growth behavior and pathogenicity for four distinct West African lineages in arthropod (Ap61) and primate (Vero) cell lines, and in mice. We found that genetic differences, as well as viral-host interactions, could play a role in the biological properties in different WNV isolates *in vitro*, such as: (*i*) genome replication, (*ii*) protein translation, (*iii*) particle release, and (*iv*) virulence. Our findings demonstrate the endemic diversity of West African WNV strains and support future investigations into (*i*) the nature of WNV emergence, (*ii*) neurological tropism, and (*iii*) host adaptation.

## Author summary

The West Nile virus (WNV) can cause severe neurological diseases including meningitis, encephalitis, and acute flaccid paralysis. Differences in WNV genetics could play a role in the frequency of neurological symptoms from an infection. For the first time, we observed how geographically similar but genetically distinct lineages grow in cellular environments that agree with the transmission chain of West Nile virus—vertebrate-arthropod-vertebrate. We were able to connect our *in vitro* and *in vivo* results with relevant epidemiological and molecular data. Our findings highlight the existence of West African lineages with higher virulence and replicative efficiency *in vitro* and *in vivo* compared to lineages similar to circulating strains in the United States and Europe. Our investigation of four West African lineages of West Nile virus will help us better understand the biology of the virus and assess future epidemiological threats.

## Introduction

West Nile virus (WNV) is a member of the Japanese Encephalitis virus (JEV) serocomplex and is a part of the genus *Flavivirus* of the family *Flaviviridae*. The WNV is a single-stranded, positive-sense RNA virus. The genomic RNA is about 11 kilobases (kb), containing one long open reading frame (ORF) flanked by 2 non-coding regions. This ORF encodes for a polyprotein, which is processed into three individual structural (Capsid, pre-Membrane, Envelope), and seven non-structural (NS1, NS2A, NS2B, NS3, NS4A, NS4B, and NS5) proteins [1–4].

West Nile fever disease (WN fever) is caused by the WNV. WN fever in humans can range from asymptomatic infections or mild acute febrile illness, to neurological diseases including meningitis, encephalitis, and acute flaccid paralysis [5–7]. WNV’s host range is extensive: it has been detected in over 65 species of mosquitoes and ticks, 225 species of birds, and 29 different animals [8,9]. A human vaccine or specific antiviral treatment for WN fever is currently unavailable.

WNV was first discovered and isolated from the blood of a woman suffering from febrile illness in 1937 in Uganda [10]. Cases of WN fever were documented in Israel and Egypt in the early 1950s, France in the 1960s, and South Africa in the 1970s [11]. The global awareness of WN fever increased in the 1990s, as sporadic and major outbreaks occurred, primarily in the Mediterranean Basin and occasionally in Europe [5]. In 1999, WNV unexpectedly emerged in New York City, signifying the first confirmed incidence of WNV in the Western Hemisphere. Since then, WNV has spread throughout the Americas, causing over 20,265 cases of neurological disease and 1,783 case fatalities in humans and even higher rates of mortality among birds in the United States [12–16].

Meanwhile, WNV continued to spread and cause WN disease and encephalitis in Europe, Asia, and Oceania [17]. In the 1990s, the largest outbreaks occurred in Romania in 1996 [18], and Russia in 1999 [19], with 17 and 40 human fatalities, respectively. In the 21^st^ century, emergences of WN fever and encephalitis have been reported in Europe [20], with a hallmark human neurological outbreak in Greece in 2010 [21], and several noteworthy outbreaks in Italy [22–24], Hungary [25] and Serbia [26].

WNV is biologically diverse; up to nine lineages have been proposed [27–30]. However, most human outbreaks of WN encephalitis have been attributed to lineages 1 and 2. Lineage 1 is globally spread and exists in distinct clades. Clade 1a comprises of strains isolated from Europe, Africa, and the Americas. Clade 1b, also referred to as Kunjin virus, has been restricted to Oceania. Major outbreaks in Europe, Africa, and the Americas with neurological diseases are caused by strains belonging to lineage 1, with an exception to clade 1b where neurological disease is rarely reported [12,31,32].

Lineage 2 was exclusively reported in Africa up until 2004, until it was isolated from humans and bird populations in Hungary, Greece, and Italy [4,21,23,33]. Lineage 2 was also considered to be less pathogenic than lineage 1, until it caused severe disease in South Africa and encephalitis among birds and humans in Europe [4,21,23,33,34]. Both lineages include strains with varying degrees of neuroinvasiveness in humans [35].

Besides lineages 1 and 2, there are lineages that are less widespread. Lineage 3, also referred to as Rabensburg virus, was repeatedly isolated in the Czech Republic [36–38]. Lineage 4 has been isolated and reported from Russia [39]. The 5^th^ lineage was isolated from India, and is often identified as a distinct clade of lineage 1 (clade 1c) [40]. A putative 6^th^ lineage, based on a small gene fragment, has been described from Spain [27,41].

Koutango virus (lineage 7) was initially classified as a different virus, but is now a distinct lineage of WN virus [31,42]. Lineage 7 strains were isolated from ticks (this study) and rodents, a rare feature among WN virus lineages [4]. The Koutango strain virus has also been shown to have a higher virulence than the lineage 1a strain “NY99” in mice [43,44]. Although there was a report of an accident where a Senegalese lab worker was symptomatically infected with the Koutango strain, a natural human infection has yet to be confirmed [45]. Additionally, a new lineage (putative lineage 8) of WNV was isolated from *Culex perfuscus* in Kedougou, Senegal in 1992 [4]. Finally, a putative 9^th^ lineage, or sublineage of lineage 4, was isolated from *Uranotaenia unguiculata* mosquitoes in Austria [27].

Despite the presence of lineages 1, 2, 7 (Koutango) and a putative 8^th^ lineage circulating in Africa [4,46,47], WNV has had minor impact on human health. Sporadic outbreaks were observed in several African counties [48–50], with lower frequencies of neurological disease than that reported from outbreaks in the USA [51,52]. For example, Senegal has never had a major outbreak of WN fever, but was the source of several endemic genotypes that were identified and sequenced. Moreover, in Senegal, WNV antibody seroprevalence has been around 80% in sampled humans, horses, and birds [53–57].

A recent study on the vector competence of African WNV lineages demonstrated that local mosquito populations lack efficient transmission of WNV [4]. Besides vector competence—*i*.*e*. intrinsic genetic variations among lineages—host adaptation, movement of host populations, climate and ecological factors could play a role in viral replication, virulence, and the outcome of infection. The N-linked glycosylation site of the envelope protein may be associated with differences observed in: (*i*) WNV neuroinvasiveness in mice, (*ii*) viral replication, and (*iii*) transmission of WNV in mosquitoes [4,58–60]. In this regard, Senegal has been a focal point in the studies of WNV virus, where multiple lineages of WNV are co-circulating endemically, but whose biology remains poorly understood.

To address these questions, we analyzed complete coding regions (polyproteins) of four different lineages circulating in Senegal and West Africa. Using additional WNV sequences from Genbank, we performed a phylogenetic analysis using the complete polyprotein sequences of the viruses and investigated sites for positive selection. We also analyzed the biological properties of these 4 WNV lineages using *in vitro* and *in vivo* models. Ultimately, understanding the relationships among ecological and genetic differences will ameliorate our understanding of WNV emergence, epidemiology, and its maintenance in nature.

## Results

### Full-length polyprotein sequencing

In this study, three complete polyprotein genes from Senegal isolates were sequenced: ArD76986, ArD96655, and ArD94343 (Table 1). These novel sequences are representative of lineages 1, 7 (Koutango) and 8 (putative), respectively. The lineage 1 and lineage 8 strains were isolated from *Culex* mosquito species, while the lineage 7 strain was isolated from a tick species.

**Table 1:** Strains of West Nile virus used in this study.

Strain	Lineage	Place of isolation	Year of isolation	Isolation source	Number of passages	Passage history^a^	Accession number
ArD76986	1	Senegal	1990	*Culex poicilipes*	10	Ap4NBM3Ap3	KY703854
B956	2	Uganda	1937	Human	11	Ap4NBM3Ap4	AY532665
ArD96655	7/Koutango	Senegal	1993	*Rhipicephalus guihoni*	8	Ap4NBM3Ap1	KY703855
ArD94343	8	Senegal	1992	*Culex perfuscus*	12	Ap4NBM3Ap5	KY703856

^a^ Ap4NBM2Ap4 is equivalent to 4 serial passages in Ap61 (Ap) followed by 2 passages in newborn mice (NBM) followed by 4 serial passages in Ap61.

**Fig 1:**
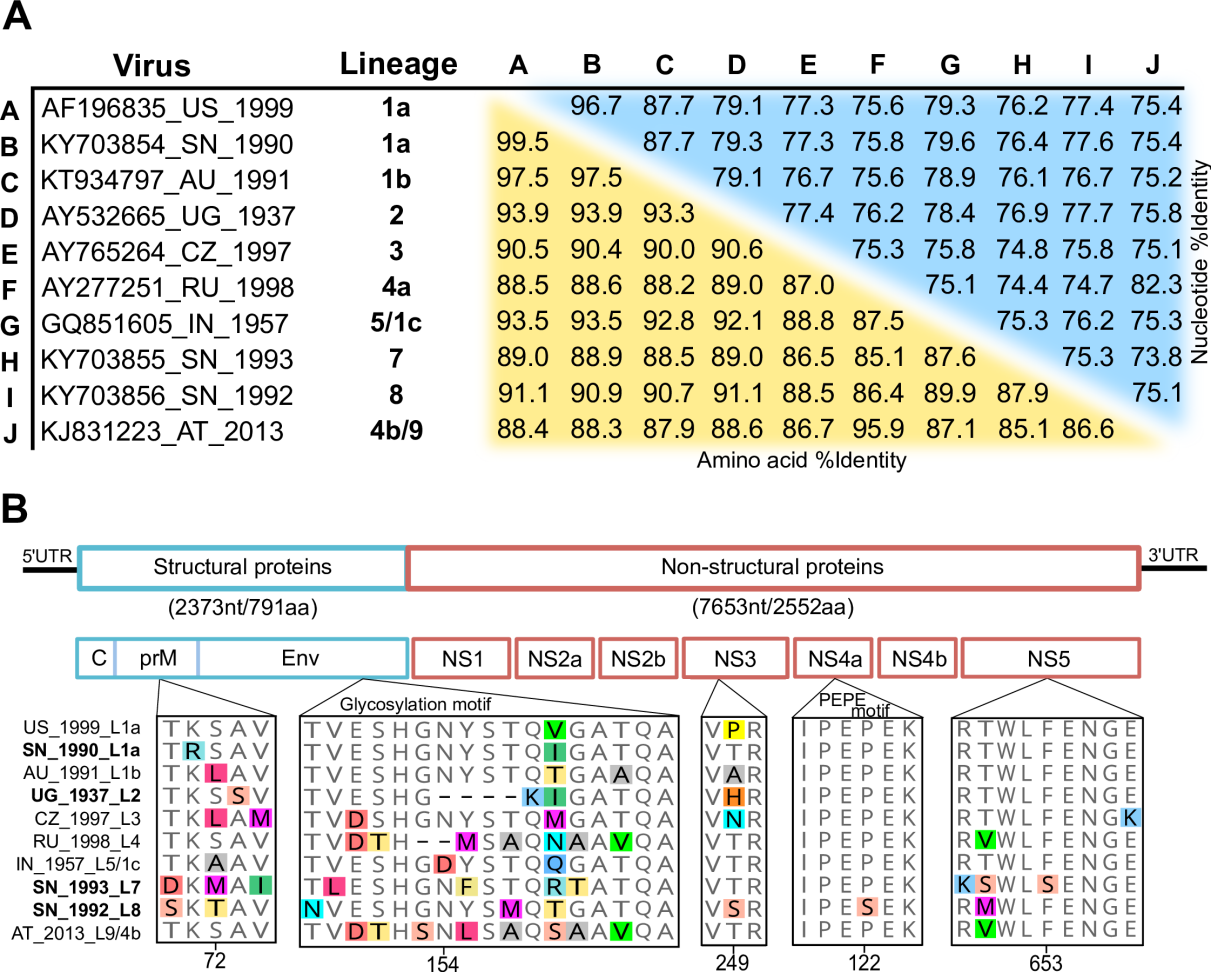
The genetic diversity of the West Nile virus lineages. A) Pairwise percent identity between nucleotide (blue) and amino acid (orange) sequences of the polyprotein. Sequences are labeled in the following format: accession number, 2-letter country code, and year of isolation. B) Genomic structure of West Nile virus with genes labeled. Alignments of known virulence motifs are shown. Codons of special interest are labeled by their position at each individual protein sequence and not by their position in the polyprotein. Sequences are labeled by country, year of isolation and phylogenetic lineage.

Acknowledging previous works that have reconstructed the evolutionary history and those that have characterized novel isolates and lineages of WNV, we included seven additional complete ORF sequences to compare differences at the gene and protein level (Fig 1). Among representative sequences, the average nucleotide pairwise identity is 77.6% (s.d. = 4.1%) and the amino acid average pairwise identity is 90.1% (s.d. = 3.3%). When comparing individual sequences, the NY99 strain (Accession number: AF196835, lineage 1a, United States 1999) shared a 99.5% pairwise identity to ArD76986 (Accession number: KY703854, lineage 1a, Senegal 1990) at the amino-acid level (Fig 1A). The sequence diversity of endemic WNV lineages in Senegal (SN) is notable, as the lineage 1 strain (ArD76986) was 88.9% and 90.9% identical at the amino-acid level to the ArD96655 (Accession number: KY703855, lineage 7, SN 1993) and the ArD94343 (Accession number: KY703856, lineage 8, SN 1992) strains respectively. Between the lineage 7 and lineage 8 strains, the amino-acid pairwise identity was 87.9% (Fig 1A).

The 1937 WNV isolate of strain B956 (Accession number: AY532665, lineage 2, Uganda 1937) is of particular interest, as it is the oldest clinical isolate available with a complete ORF sequenced. Amino acid pairwise identity was 93.9% to the NY99 strain sequence and to the lineage 1a (SN) sequence, 89% to the lineage 7 strain sequence and 91.1% to the lineage 8 strain sequence. At the nucleotide level, the lineage 2 strain (UG) had a pairwise identity of 79.1% to the NY99 sequence, 79.3% to the lineage 1a strain (SN) sequence, 76.9% to the lineage 7 strain sequence and 77.7% to the lineage 8 strain sequence. Additionally, B956 contains a 12 base pair deletion at nucleotide position 1,331, corresponding to the WNV envelope glycosylation site.

We compared published sequences and published works to identify whether mutations that have been shown to influence WNV virulence and replication were present in the newly sequenced open-reading frames. For each strain, we discovered amino acid changes that were associated to a phenotypical change and many additional mutations with unknown consequences (Fig 1B). For example, the 22^nd^ and 72^nd^ codon sites of the pre-membrane protein (prM) have been shown to play a role in enhancing the virulence and particle secretion in WNV [61]. At this site, we found alterations in the lineage 7 strain (SN_1993_L7), and the lineage 8 strain (SN_1992_L8).

Another example is the glycosylation site found in the 154-156^th^ positions of the envelope (Env) protein, which is considered a virulence factor [62]. We found that the lineage 1 strain from Senegal (SN_1990_L1a), the Kunjin strain (AU_1991_L1b), the NY99 strain (US_1999_L1a) and the lineage 8 strain (SN_1992_L8) harbored the NYS motif while other strains had variations or deletions in this locus. Next, the 249^th^ codon position of the NS3 protein [63], the helicase protein, was found to increase viremia and virulence in birds, and could play a role in other hosts. We observed several variations in our data at the 249^th^ codon position (Fig 1B).

Additionally, changes in the highly conserved ^120^P-E-P-E^123^ region of the NS4A protein can attenuate or even impair virion replication and release [64], which we found present in the lineage 8 strain. Finally, a mutation in the NS5 protein, serine (S) to phenylalanine (F) at the 653^rd^ position in the NS5 protein, is associated with an increased resistance to interferon [65], a mutation that is shared by the lineage 7 strain (SN_1993_L7) (Fig 1B). We also found several synonymous changes in positions corresponding to known virulence motifs, such as variability in the third codon site position (the wobble base) during the translation of serine (S) at the 156^th^ codon site. We also investigated sites within the NS2A [66], NS4B [67], and additional sites within the NS5 region that are known to impact on infectivity and virulence [65], but no mutations were present in our sequences.

### Phylogeny of West Nile virus

The phylogenetic analysis revealed a similar topology to the ones obtained from previous maximum likelihood trees [27,40,42,68,69]. Currently, up to 9 distinct lineages have been suggested.

**Fig 2:**
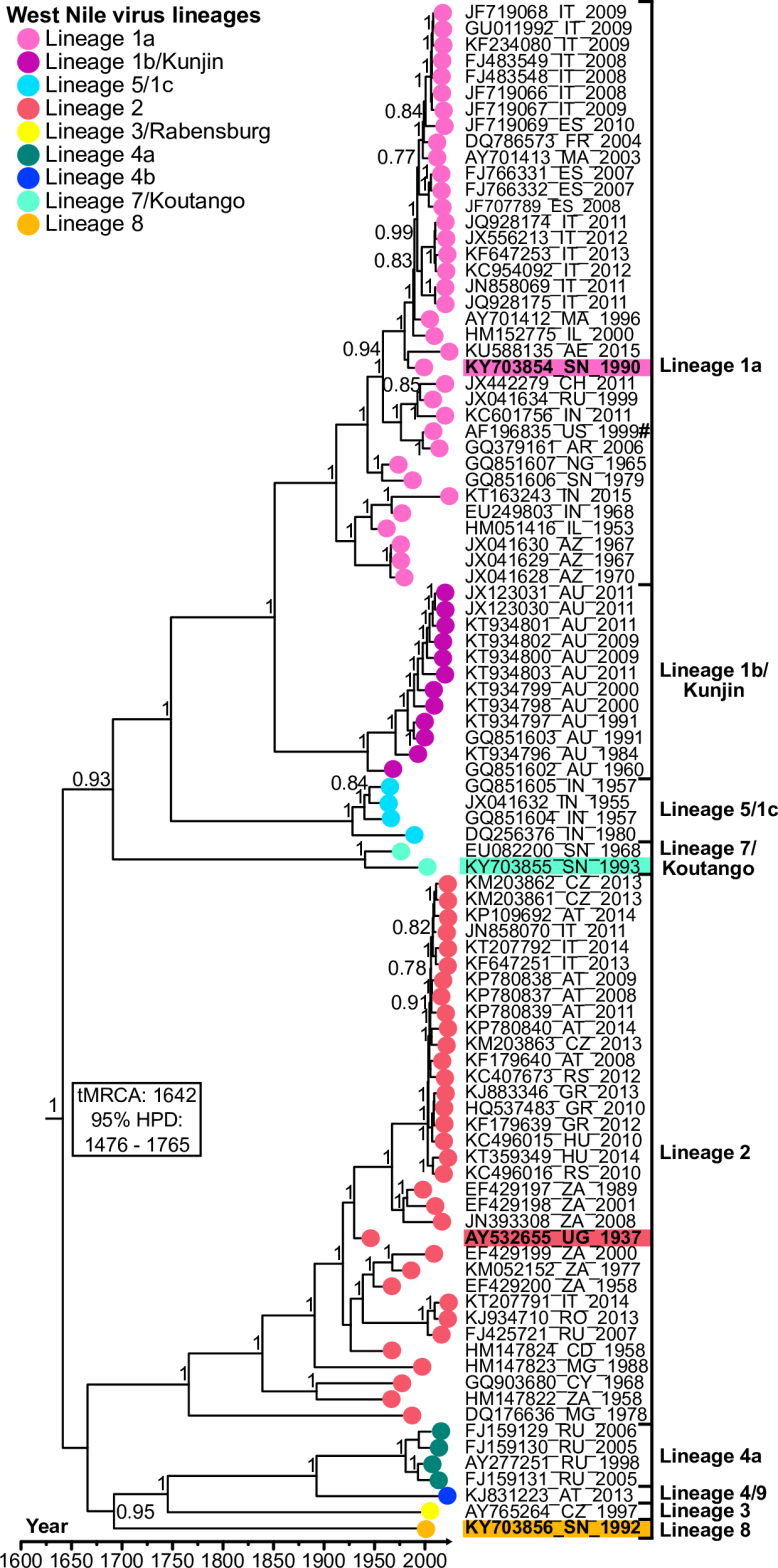
Bayesian maximum clade credibility tree estimating the phylogenetic relationships of West Nile virus. Tree nodes with a posterior probability greater than 0.7 are displayed. Tree tip nodes are colored by proposed lineage and for visual clarity. For each sequence, the two-letter code representing a country of isolation is included in the sequence label. Branches are scaled in years before 2015. ^#^ NY99 strain.

A total of 95 sequences, including 3 novel polyprotein sequences from Senegalese isolates (Table 1), were used to estimate a maximum-likelihood tree with FastTree (S1 Fig) and a very similar relaxed clock Bayesian maximum-clade credibility (MCC) tree (Fig 2), summarizing the MCMC runs with BEAST. The MCC tree was scaled to time (years) and branch tip-nodes were colored to identify previously classified lineages [27]. Here, the time to the most recent common ancestor (tMRCA) with its corresponding 95% highest posterior density (HPD) interval for WNV was estimated in the unit of years. The tMRCA of WNV is predicted to have originated in the late 16^th^/early 17^th^ century (95%HPD: 1476–1765), a major split that diverges lineages 1, 5 and 7 from lineages 2, 3, 4, 8, and 9. Both lineage 1 and 2 show multiple introductions into Europe and other *New World* countries. Additionally, we see that lineages 1, 2, 7, and 8 have been isolated in West Africa, yet only lineages 1 and 2 have emigrated.

### Growth kinetics

**Fig 3:**
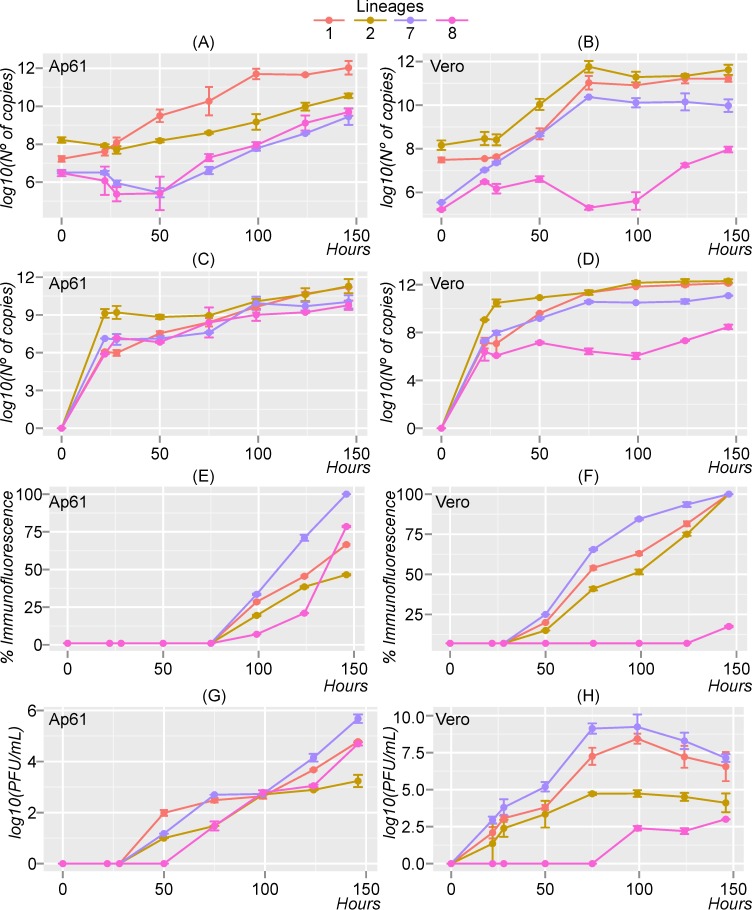
Growth kinetics of West African West Nile virus strains. The strain lineage label is in reference to the strains in Table 1. Figs A-D show the amount of viral RNA equivalents isolated from cells (A and B) and supernatant (C and D) (log_10_ of RNA copy number), the percent (% immunofluorescence) of cells infected (E and F) and the number of infectious viral particles (G and H) (log_10_PFU/ml) over a 146-hour post-infection time period. The experiments were performed with Ap61 cells (left column) and Vero cells (right column). The error bars indicate the range in values of two independent experiments.

Infection, viral proliferation, and virulence in each cell type were measured by 4 different tests over a 146 hours post-infection period: quantitative reverse transcriptase PCR (qRT-PCR) of the lysed cell fracture to measure genome replication (Fig 3A and 3B), qRT-PCR of the supernatant fraction to detect genome replication dynamics (*i*.*e*., total number of particle release) (Fig 3C and 3D), immunofluorescence staining of the cells to visualize the infectivity of cells and estimate protein translation efficiency (Fig 3E and 3F), and plaque assays to determine the amount of infectious viral particles (PFU/ml) from the supernatant fraction (Fig 3G and 3H). Using Ap61 and Vero cells, our goal was to replicate the biology of WNV in a mosquito vector and its vertebrate host.

We found that African lineages have different growth dynamics in mosquito and mammalian cell lines. In *Aedes pseudoscutellaris* cells, growth dynamics were similar for all lineages, (Fig 3, left column) where all lineages exhibited successful replication and generation of infectious particles. In Vero cells (Fig 3, right column), lineages 1, 2, and 7 showed exceptional growth, with lineage 2 strain exhibiting the highest replication and particle release capabilities, and lineage 7 strain having exceptional translational dynamics and highest PFU/ml during the infection interval.

We observed cell-specific growth differences among different WNV strains. For example, Fig 3A and 3B showed differences in genome replication dynamics in the cells with respect to host cells. Interestingly, lineage 1 strain had higher genome replication in Ap61 cells (*p*-value ranging from 2.22x10^-16^ to 0.002) while the lineage 2 strain had higher genome replication in Vero cells (statistically comparable). Lineage 8 showed a lower significant replication profile in Vero cells (*p*-value ranging from 8.81x10^-13^ to 0.031). Furthermore, differences in growth at T_0_ further supports that WNV lineages could have a preference to a specific cellular environment. The rate of viral attachment, entry and replication initiation can all depend on the genetics of the infecting strain [70].

We estimated the total number of released particles at different times post infection by measuring the WNV RNA copy number in the cell supernatant. All tested lineages had comparable genome copy numbers in Ap61 supernatants (Fig 3C). However, we found a significantly higher copy number of total particles released for the lineage 2 strain at 22, 28, and 50 hours post-infection (hpi) in both *in vitro* models (*p*-value ranging from 2.22x10^-16^ to 0.023). Lineage 8 strain showed significantly lower genome copy numbers in Vero supernatants (*p*-value ranging from 8.81x10^-13^ to 0.031).

Next, we approached differences in protein translation efficiency between lineages by detecting viral proteins using an immunofluorescence assay (IFA) (Fig 3E and 3F). The lineage 7 strain displayed more efficient protein translation in both cells (*p*-value ranging from 3.98x10^-13^ to 0.011), while lineage 8 strain had significantly lower levels of protein translation in Vero cells. Nevertheless, the translation rate in lineage 8 increased significantly from T_124-146_ in Ap61 cells. We also noticed a delay on translation detection in both cells, with no detectable protein production until T_99_ hours (Fig 3E) and T_50_ hours (Fig 3F) respectively.

To quantify the infectious particles of different WNV strains, we used plaque assays to estimate the amount of infectious viral particles (PFU/ml) in the supernatant fractions. In Ap61 cells, we found a similar profile of infectious particles production for all lineages, with significant higher rates at 124 hpi and 146 hpi for the lineage 7 strain (*p*-value ranging from 2.22x10^-16^ to 0.028) (Fig 3G). In Vero cells, lineage 1 and lineage 7 strains had higher number of PFU/ml, while lineage 2 had an intermediate profile and lineage 8 had the lowest amount of infectious viral particles, with significant differences from 28 to 124 hpi (*p*-value ranging from 2.22x10^-16^ to 0.049) (Fig 3H).

**Fig 4:**
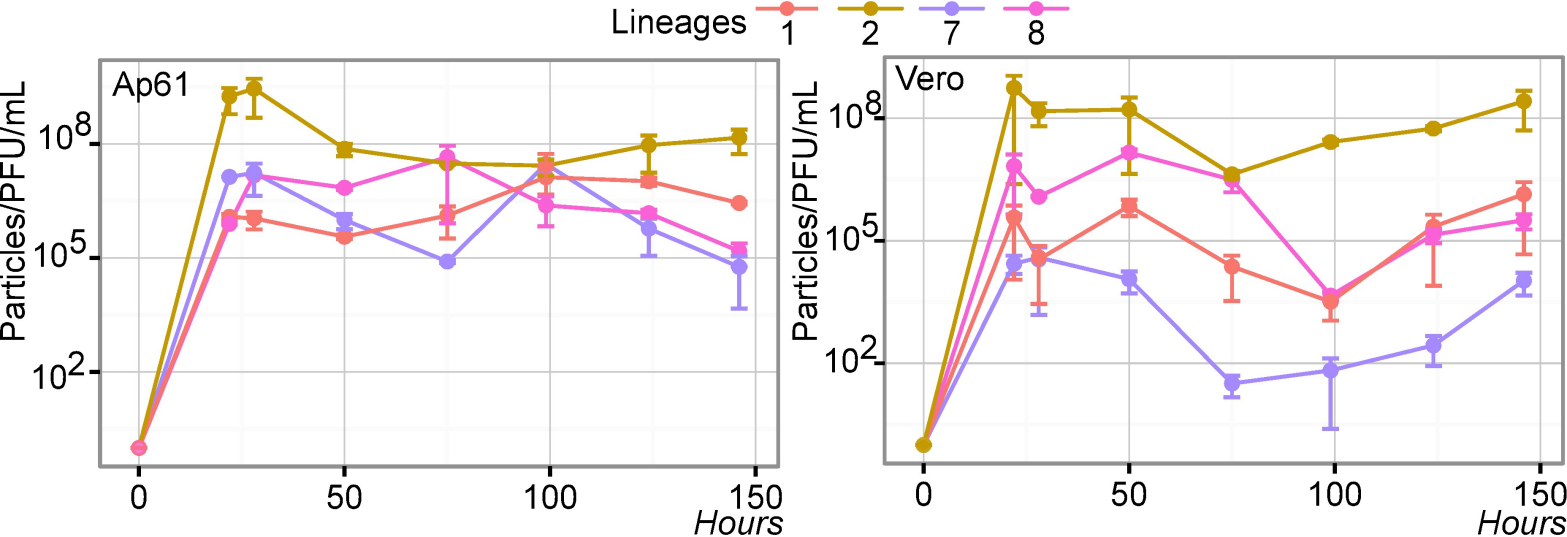
Replication Efficiency of West Nile virus *in vitro*. Replication efficiency of West African strains in Ap61 and Vero cell lines over 146 hour post-infection period. The error bars indicate the range in values of two independent experiments.

Finally, we approximated the replication efficiency by finding the ratio of the number of virions released in the supernatant–particles that completed the infectious cycle–divided by the number of plaque forming units (PFU) [71–73]. We estimated the ratio for each strain in each cell and found significant differences in replication efficiency (*p*-value ranging from 5.49x10^-16^ to 0.0223) (Fig 4). There are some consistencies with Fig 3, where the lineage 7 strain was the most efficient and the B956 strain was the least efficient *in vitro*. Lineage 1 and lineage 7 strains seem to be more cell-specific; both replicated less efficiently in Ap61 cells.

### Virulence and survival of West Nile virus *in vivo*

To determine the virulence of WNV strains (Table 1), we challenged five- to six-week-old mice with three different viral doses and observed their overall survival for 21 days. Depending on the strain and dose used, several mice developed clinical disease and died (Table 2). Clinical signs included tremors, reduced activity and reluctance to move, hind leg paralysis and closed eyes. The PBS-inoculated control groups exhibited no signs of disease throughout the experiment.

**Table 2:** Mice mortality and virulence of West Nile virus *in vivo* of 5- to 6-week-old Swiss mice observed for 21 days.

Strain	Lineage	Viral dose (PFU)	dead/total (mice)	%Mortality	AST^a^(days)
ArD76986	1	100	0/12	0	-
		1000	6/12	50	16.4
		10000	5/12	42	16.7
B956	2	100	1/12	8	19.7
		1000	7/12	58	14.3
		10000	3/12	25	18.7
ArD96655	7	100	8/8	100	5.5
		1000	12/12	100	10.8
		10000	12/12	100	9.2
ArD94343	8	100	0/12	0	-
		1000	0/12	0	-
		10000	1/12	8	19.7

^*a*^ AST (Average survival time)

**Fig 5:**
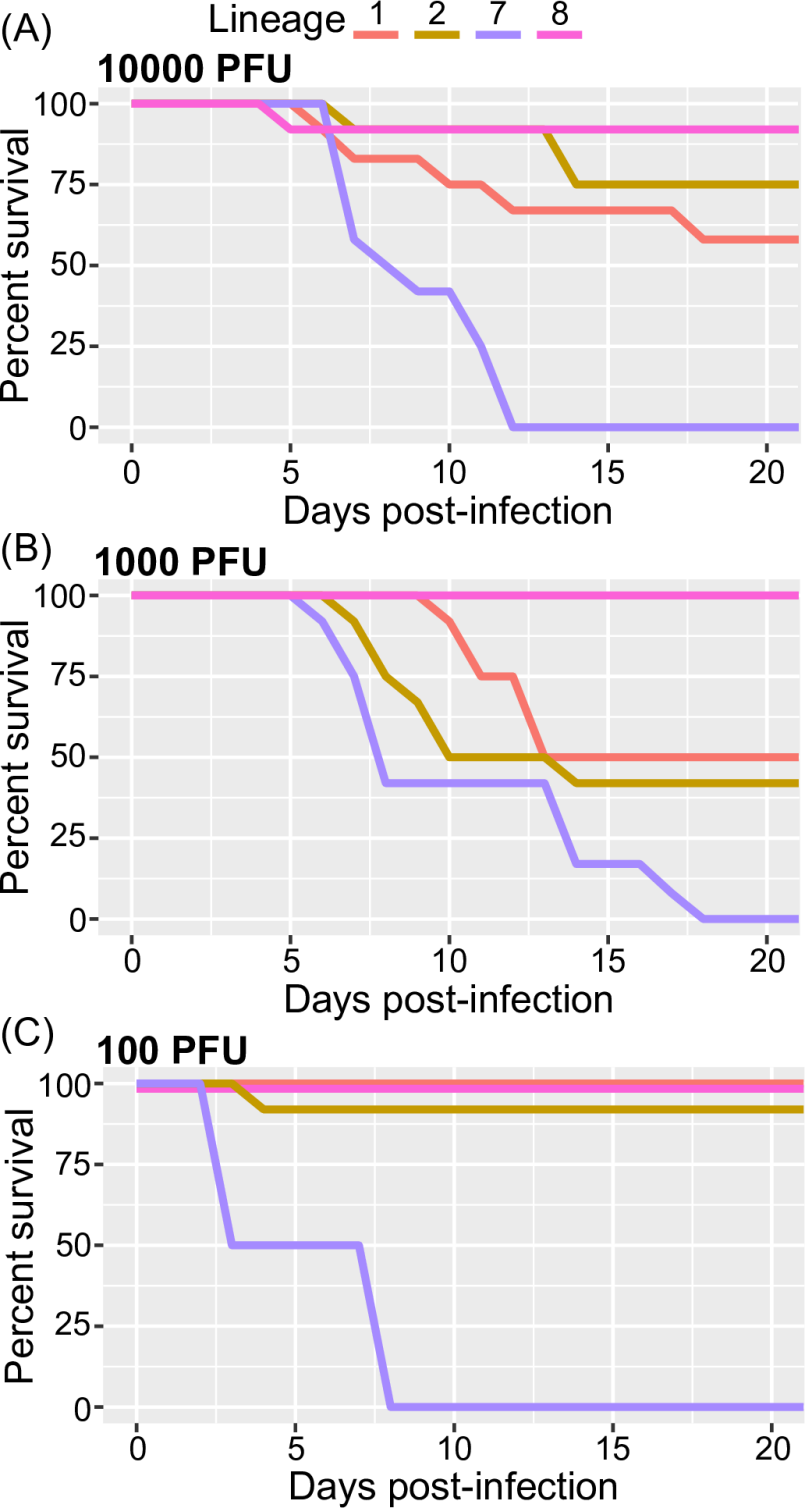
Survival curves of 5- to 6-week-old mice following intraperitoneal infection with (A) 10,000, (B) 1,000 and (C) 100 PFU. Mice were monitored daily for 21 days. Except in (C) when comparing lineage 8 and lineage 1, all survival curves were significantly different (Wilcoxon rank sum test, *p-*values < 0.05).

The lineage 7 strain was the most virulent of the strains at all administered doses (Wilcoxon rank sum test, *p-*values < 0.05). In fact, the lineage 7 strain induced the shortest survival time compared to the other strains and always resulted in 100% mortality in every experiment (Fig 5 and Table 2). Interestingly, in most cases, mice inoculated with the lineage 7 strain died without showing any clinical signs.

Comparatively, mice inoculated with the lineage 1 and lineage 2 strains usually showed signs of disease at least 1 day before dying. However, lineage 8 showed no virulence (100% survival) at 100 and 1000 PFU doses (Fig 5B and 5C). In fact, only one mouse mortality was observed at 10000 PFU (Fig 5A and Table 2).

### Selection

To determine the evolutionary pressures acting on the WNV ORF, we estimated the ratio of nonsynonymous (*d*N) to synonymous (*d*S) substitutions per codon site (where *d*N—*d*S > 0, signifies positive selection) using 95 sequences, which represent all investigated WNV lineages. Our investigation on selection regimens acting on all WNV complete ORF sequences—with the FUBAR method—revealed 3313 well supported (posterior probability ≥ 0.9 and Bayes Factor < 3.0) sites under purifying selection (S1 Table and S2 Fig). However, we found 95 statistically significant sites (*p*-value ≤ 0.1) under diversifying episodic selection (S2 Table and S3 Fig), using MEME method.

## Discussion

Despite the presence of at least four different lineages in West Africa, there has never been a major outbreak, nor a large frequency of encephalitic cases connected with WNV. The lack of a WN disease “burden” within Senegal could suggest that WNV is endemic, which could explain the high seroprevalence, and therefore, few susceptible hosts [54]. However, the threat of WNV emerging to places where the population’s seroprevalence is much lower or even naive is a serious concern. Avian migratory routes could have played a role in the emigration of WNV strains from Africa [53], and for other African-borne arborviruses such as Usutu virus [74]. The extensive genetic diversity (Fig 1A) and broad host range of WNV [8,9] could have also contributed to its global dissemination (Fig 2), as certain mutations have been previously prosecuted with lineage 1’s entrance in the United States [32] and lineage 2’s emergence in Europe [75]. As a consequence, several groups have investigated how specific genetic changes and selective pressures within the WNV ORF can affect the phenotypical behavior of a WNV strain.

In our study, the growth kinetics of the different West African WNV lineages were explored in *Aedes pseudoscutellaris* (Ap61) and African green monkey kidney cells (Vero) to reflect infection dynamics in two common classes of WNV hosts (insect vector and primate) (Figs 3 and 4). The virulence of these lineages in mice was also analyzed (Fig 5 and Table 2). We found that these 4 West African lineages have significant differences in their ability to proliferate in our tested cell lines and their degree of virulence in mice (Figs 3, 4 and 5). We also explored how our *in vitro* and *in vivo* results could be explained by their evolutionary (Fig 2) and individual genetic variations (Fig 1).

In agreement with other viruses that use alternate hosts (vertebrate-arthropod-vertebrate) and cause acute infections, we found that the majority of WNV codon sites are undergoing purifying selection [76]. Nevertheless, some of the significant episodic diversifying sites that we found are related to virulence, like the 444^th^ and 446^th^ codons in polyprotein gene (154-156^th^ positions of the Env protein) that encode the N-glycosylation motif (NYS). This site is present in many lineage 1 strains, some neuroinvasive lineage 2 strains [34,77], and the lineage 1 and 8 strains from this study (Fig 1B). We found significant diversifying selection acting on these codon sites in 3% and 24% in all of the WNV lineages, respectively (S1 Table). These episodic non-conservative changes could have resulted in the loss of the N-linked glycosylated site motif, which is related to less efficient replication in *Culex* cells [59] and better replication in *Aedes albopictus*cells [60]. This N-linked site is also associated with neuroinvasiveness in mice [62]. Second, we found that 1754^th^ codon in polyprotein (249^th^ in NS3) was under diversifying selection (ω = 33.1) 13% of the time and under purifying selection (ω = -0.91) 87% of the time (S1 Table) in our WNV dataset. As this site was discovered to increase viremia and virulence in birds [63], further experiments in avian cell lines should be explored to see if our discovered substitutions effect replication in avian hosts and transmission dynamics.

Although no significant diversifying selection was observed on the cleavage site in the NS4A protein (^120^PEPE^123^ motif), we did discover the P122S substitution in the lineage 8 strain (Fig 1B). Crucially, induced mutations in this motif are related to low rates of replication and protein production in Vero cells [64], which we expected and observed for the lineage 8 strain *in vitro* (Fig 3B, 3D and 3F), and could help explain its low virulence *in vivo* (Fig 5). In general, we observed little change in viral replication and protein production between West African strains in Ap61 cells (Fig 3A, 3C and 3E). This could suggest that the conservation of PEPE motif may have a lesser role in replication in mosquito cells (lineage 8) or that the strains may have been “pre-adapted” prior to our experiment.

The lineage 7 strain has the S653F NS5 mutation that is associated with an increased resistance to interferon [65], which could help explain its phenotypical virulence *in vitro* and *in vivo* (Figs 1B, 3, 4 and 5). However, because Vero cells are known to be interferon-deficient, we could not associate this mutation to our *in vitro* results for lineage 7 in Vero cells (Fig 3, right column). Nevertheless, all three West African strains contained a non-synonymous change in a locus that was previously explored by site-directed mutagenesis experiments (Fig 1B). Interestingly, we also detected synonymous changes in the “wobble” base position of the codons in “sites of interest”. However, our knowledge of how synonymous changes impact infectivity, virulence, and replication of WNV is still limited.

As previously described, lineages 1 and 2 originated in Africa and emerged as a *New World* pathogen over the last 60 years [68]. Lineage 7 could be following a similar path; besides Senegal, it has been detected in Somalia, Gabon and possibly in Italy [78–80]. Our study supports that there are other lineages besides 1 and 2—such as lineage 7—that can exhibit high virulence in mice and efficient replication in mammalian cells (Figs 3, 4 and 5). This high virulence of lineage 7/Koutango strains in mice has been explored in two other studies, where the high virulence is suggested to be a result of delayed viral clearance and a weak neutralizing antibody response [43,44]. All three doses tested resulted in 100% mice mortality (Table 2), which agreed with previous results. The differences in average survival time and mortality rates compared to previous studies, could be explained by differences in the passage history of viral strains, and the age of the infected mice [81].

The Senegalese lineage 1 strain exhibited moderate virulence in mice (Fig 5) and caused comparably less mortality than the NY99 strain when compared to similar studies [44]. Differences in neuroinvasive potential and virulence among lineage 1 strains has been reported and could be explained by genetic differences [35]. Alternatively, lineage 8 showed poor growth capabilities in Vero cells (Figs 3 and 4) and almost no virulence in mice (Fig 5 and Table 2), suggesting that it may be restricted to vertical transmission or is species restrictive. Lineage 8 was described to have a similar phenotype to Rabensburg virus (lineage 3, Czech Republic 1997). Moreover, growth kinetics and vector competence studies revealed poor growth of the Rabensburg virus in mammalian cell lines and low virulence in mice [36,82]. This similarity could indicate that both lineage 8 and the Rabensburg strain may be restricted in host range and are also maintained in nature through vertical transmission. Investigating the vector competence of lineage 8 in different arthropod species (*i*.*e*. *Culex*, *Aedes*, and tick species) could lead to a better understand the transmission dynamics and maintenance cycles of WNV in nature. The low virulence phenotype of the lineage 8 strain could also be a factor for its consideration as a potential vaccine candidate for West Nile fever.

Further studies could complement our analysis, particularly, on other factors that could explain differences in WNV host and disease dynamics. Exploring variations in codon usage bias could also help explain biological differences [83], as distinct lineages have shown different degrees of natural selection and mutational bias. Site-directed mutagenesis studies may also help explain how strain-specific mutations, both synonymous and non-synonymous, could explain deviations in replication efficiency and virulence for our *in vitro* and *in vivo* results. For example, future studies in cell lines with interferon may help clarify the impact of the S653F NS5 mutation for the lineage 7 strain. Additionally, the flavivirus 5’ and 3’ untranslated regions (UTR) can affect replication and translation; certain mutations in these regions can cause complete viral attenuation [84–87]. Unfortunately, we could not investigate their impact in this study, as the majority of WNV UTR’s were publically unavailable.

Taking everything into account, especially differences in sequences, growth dynamics and virulence *in vivo*, the West Nile virus is a pathogen with the capability to cause severe epidemics anywhere in the globe. As complete genome sequences including the 5’ and 3’ UTR regions are currently being generated, this could lead to future studies focused on *in vivo* transmission and growth dynamics. As additional strains of WNV are characterized, monitoring the global diversity and distribution will aid in threat assessment and epidemiological modeling if future outbreaks are to occur.

## Materials and methods

### Cell lines

Two cell lines have been used for virus cultivation and growth kinetics. Ap61 cells (*Aedes pseudocutellaris*) were grown in L15 (Leibovitz’s 15) medium (10% heat-inactivated fetal bovine serum [FBS], 1% penicillin-streptomycin, 0.05% amphotericin B [Fungizone] (GIBCO by life technologies; USA) and 10% tryptose phosphate (Becton, Dickinson and Company Sparks, USA) and incubated at 28°C without CO_2_. Vero cells (African green monkey kidney epithelial cells*; Cercopithecus aethiops*) (obtained from Sigma Aldrich, France) were grown using the same medium without tryptose phosphate and CO_2_. Furthermore, PS (Porcine Stable kidney cell line, American type Culture Collection, Manassas, USA) cells were grown in same conditions than Vero cells and have been used for plaque assay.

### Virus strains

The virus strains used in this study corresponding to lineages 1, 2, Koutango (lineage 7) and 8 were described in Table 1. The virus stocks were prepared by inoculating *Aedes pseudoscutellaris* (Ap61) continuous cells lines for 4 days. The infection status was tested by immunofluorescence assay (IFA), real-time RT-PCR (Reverse Transcriptase-Polymerase Chain Reaction) and plaque assay. The supernatant of infected cells were aliquoted, frozen at -80°C, and used as viral stocks for growth kinetics.

### Phylogenetic analyses

A total of 862 complete WNV polyprotein gene sequences with country and year of isolation data were available and initially downloaded from Genbank for this study. A large number of sequences were from the Americas and formed a monophyletic group of lineage 1a comprising 770 sequences. To reduce computer-processing requirements while maintaining the authenticity of our results, we removed all lineage 1a sequences except for a single representative sequence denoted “NY99” (accession number: AF196835). With the addition of 3 new sequences, a total of 95 sequences were aligned using Muscle v3.8.31 [88] and manually curated using Se-Al v2 [89]. For Fig 1, the available complete polyprotein sequences representative of WNV diversity (excluding lineage 6, which there is only a partial sequence available) were included to compare genetic percent identities.

Likelihood mapping analyses for estimation of data quality were performed using Tree-Puzzle (Quartets ranged between 10,000 and 40,000) [90,91]. For each alignment we performed recombination screening (RDP, GeneConv, Chimaera, MaxChi, BootScan and SiScan) in RDP4.61 [92].

The Bayesian phylogenetic analysis was performed using Bayesian Inference (BI) using a general time-reversible with gamma-distributed rate variation and invariant sites model (GTR+Γ+I), as selected by Akaike's information criterion (AICc) in jModelTest 0.1 [93]. The evolutionary analysis was conducted assuming a relaxed Gamma clock and GMRF Bayesian Skyride coalescent tree prior. We then employed a Bayesian MCMC approach using BEAST v1.8.4 and performed five independent MCMC runs with up to 100 million generations to ensure the convergence of estimates. Trees were summarized in a maximum clade-credibility tree after a 10% burn-in [94] and used Tracer (http://beast.bio.ed.ac.uk/Tracer) to ensure convergence during MCMC by reaching effective sample sizes greater than 100.

To reduce the number of sequences from the original 862 downloaded from Genbank, a maximum likelihood tree was estimated using FastTree v2.1.7 [95] after identical alignment and curating methods. FastTree was run using GTR+Γ+I nucleotide model with 2000 Γ-rate categories, exhaustive search settings, with 5000 bootstrap replications using the Shimodaira-Hasegawa (SH) test. The analysis was repeated for the dataset of 95 sequences to compare tree topologies inferred by the Bayesian approach (S1 Fig). All alignments referred to in this manuscript can be found at https://github.com/caiofreire.

### Growth kinetics

To perform this study and make it comparable with other studies [60,96], viral stocks were standardized in number of plaque forming units per milliliter (PFU/mL) for cell infections rather than copy numbers of genome. The growth kinetics assays were performed in 12-well plates using one plate per virus strain with one uninfected well as a negative control. Each well was seeded with 2.4x10^5^ Ap61 or Vero cells in a volume of 400 μl of appropriate medium and infected with 2.4x10^3^ PFU (plaque-forming unit) of virus in 400 μl of medium, resulting in a multiplicity of infection (MOI) of 0.01. After an incubation time of 4 hours, the medium was removed and replaced with 2 ml of new medium to set a zero point for the growth curves (T_0_). The harvesting of one well occurred at 22, 28, 50, 75, 99, 124, and 146 h post infection. Each harvest was performed as follows. Supernatants were removed and frozen at -80°C in small aliquots. Cells were washed once with phosphate-buffered saline (PBS) and then removed in 500 μl PBS. A volume of 20 μl of cell suspension was dried on a glass slide for a subsequent immunofluorescence assay as previously described [97] to measure viral proteins production. The remaining cell suspensions were frozen at -80°C.

RNA was extracted from cell suspensions and supernatants and copy numbers of genome were quantified by real time RT-PCR as previously described [98]. Infectious viral particles were measured in supernatants by plaque assay also as previously described [99]. This study was performed two times on each cell type. The initial titers of lineages 1, 2, 7 and 8 were respectively 3x10^8^, 5x10^4^, 7.5x10^6^ and 10^10^ PFU/ml. For each lineage, 2.4x10^3^ PFU were used for kinetics in mosquito and mammal cells. The ratio of particles per infectious unit in the initial viral stocks ranged from 8 to 600 [98]. Our viral stocks had a similar ratio of particles per infectious unit as that seen produced by fully infectious extracellular WN virus particles [100] and mosquito-derived replicon WN virus particles [101]. Variances in replication efficiency between studies observed during *in vitro* infection could be explained to differences in the viral strain and to the infection conditions *i*.*e*. very low MOIs (0.01), and distinct cell lines.

### Mouse infection and survival studies

Mice were produced in the Institut Pasteur de Dakar farm, located in Mbao, approximately 15 kilometers from Dakar, Senegal. After one week of acclimatization, five-to-six-week-old Swiss mice were challenged by intraperitoneal (IP) injection with 100, 1000 and 10000 PFU of WNV lineages diluted in phosphate buffer saline + 0.2% endotoxin-free serum albumin (BSA). For each lineage and dose, two independent experiments of infection were made. Each individual experiment had 4 to 8 mice. A group of mice inoculated in parallel with an equivalent volume of phosphate buffer saline + 0.2% endotoxin-free serum albumin (BSA) was maintained as a control. Mice were kept on clean bedding and given food and water *ad libitum*. Infected animals were monitored daily for first signs of encephalitis (hunching, lethargy, eye closure, or hind legs paralysis) and death throughout the 21 days after infection. All statistical inferences were calculated using the Wilcoxon rank sum test.

### Selection

To evaluate selection patterns on the complete coding sequences, we estimated the ratio of substitution rates (ω) per non-synonymous site (*d*N) over synonymous substitutions per synonymous site (*d*S) per codon sites. Briefly, sites with ω>1 are assumed to be under positive (diversifying) selection, and sites where ω<1 are undergoing negative (purifying) selection. When ω = 0, the site is undergoing neutral selection. To estimate ω, we applied three maximum likelihood methods: single likelihood ancestor counting (SLAC), fixed-effects likelihood (FEL), and internal fixed-effects likelihood (IFEL). We also investigated the presence of transient (episodic) selective pressures, using the mixed-effects model of evolution (MEME) [102] and fast, unconstrained Bayesian approximation (FUBAR) [103] approaches. For FEL, SLAC, IFEL, and MEME analyses, sites were identified as undergoing significant positive selection when *p*-value ≤0.10. For FUBAR, sites were identified as undergoing positive selection when there was a posterior probability ≥0.90. All estimations were implemented using HyPhy v2.11 [104].

### RNA extraction and quantitative real-time (qRT-PCR)

Extraction of viral RNA from supernatants was performed with the QIAamp viral RNA mini kit (Qiagen, Heiden, Germany) according to manufacturer’s instructions. For cell fractions, prior to RNA extraction, cells were lysed by serial cycles of freeze/thaw. For the detection and quantification of viral RNA, a consensus WNV real-time RT-PCR assay and corresponding RNA standard were used as previously described [98]. The real-time PCR assays were performed using the Quantitect Probe RT-PCR Kit (Qiagen, Heiden, Germany) in a 96-well plate under the following conditions: 50°C for 10 min, 95°C for 15 min followed by 40 cycles of 95°C for 15 s and 60°C for 1 min. Copy numbers of genome were calculated using Ct (Cycle threshold) and corresponding RNA standard.

### Complete polyprotein sequencing

Overlapping RT-PCRs were done to recover the complete genome. All primer sequences can be found in the S3 Table. The NS5, envelope and NS5-partial 3’UTR regions were first amplified using flavivirus consensus or West Nile specific primers [1,105,106], followed by amplification of NS3 region using designed WNV primers. The 5’ non-coding region of the genome was obtained using the 5’RACE kit (Invitrogen, Carlsbad, USA) and a designed consensus primer in the capsid protein for reverse primer. Finally, specific primers were designed according to the first sequences obtained and a second step of RT-PCR was done to obtain the complete genome.

The PCR fragments were obtained using AMV reverse transcription kit (Promega, Madison, USA) for reverse transcription and Go-Taq PCR kit (Promega, Madison, USA) for amplification. The RT conditions were set according to the manufacturer’s instructions, and the PCR conditions were as follows: 5 minutes at 95°C, 40 cycles of 1 minute at 95°C, 1 minute at 53°C, 1 to 4 minutes (according the size of the PCR product) at 72°C, and 10 minutes at 72°C. The PCR products were purified from the agarose gel using the Gel extraction kit (Qiagen) and sequenced by Cogenics (Beckman Coulter Genomics, Essex, UK).

### Immunofluorescence assay (IFA)

Infected cells at different time points were dissolved in PBS and dropped on a glass slide. After complete drying, cells were fixed for at least 20 min in cold acetone, dried again, and then stored at -20°C until staining. Staining was done with a WNV-polyclonal mouse immune ascit diluted in PBS and incubated for 30 minutes at 37°C. After washing three times with PBS, cells were incubated with the second antibody (goat anti-mouse IgG, fluorescein isothiocyanate [FITC] conjugated Biorad), diluted 1:40 and blue Evans 1/100 in PBS, for 30 minutes at 37°C in the dark. The cells were washed again three times with PBS, dried, and covered with 50% glycerol in PBS. After dehydration, examination was done by fluorescence microscopy.

### Genbank accession numbers

KJ831223, FJ159131, AY277251, FJ159130, FJ159129, AY765264, KY703856, DQ176636, HM147823, FJ425721, KT207791, KJ934710, KP780840, KP780839, KT359349, KC496016, KC407673, KF179639, KJ883346, KC496015, HQ537483, KF647251, KT207792, JN858070, KP109692, KF179640, KM203863, KP780838, KP780837, KM203861, KM203862, JN393308, EF429197, EF429198, HM147824, EF429199, KM052152, EF429200, GQ903680, HM147822, GQ851605, DQ256376, GQ851604, JX041632, GQ851602, KT934796, KT934801, JX123031, JX123030, KT934800, KT934802, KT934803, GQ851603, KT934797, KT934799, KT934798, JX041628, JX041629, JX041630, HM051416, KT163243, EU249803, KC601756, JX442279, JX041634, KU588135, JQ928175, JN858069, KF647253, KC954092, JQ928174, JX556213, JF707789, FJ766331, FJ766332, JF719069, FJ483549, FJ483548, JF719066, JF719067, KF234080, GU011992, JF719068, DQ786573, AY701413, HM152775, AY701412, GQ851606, GQ851607, GQ379161, AF196835, KY703855, EU082200, KY703854, AY532665.

## Supporting information

S1 Fig Phylogenetic inference of West Nile virus using a maximum-likelihood tree. The Shimodaira-Hasegawa values greater than 70% are shown at respective nodes. Tip labels are colored by proposed lineage. Sequences from Table 1 are labeled.
(TIF)

S2 Fig Selection regimens acting on codons of West Nile Virus polyprotein via FUBAR method. The dashed line marks neutral selection (dN-dS = 0), points above the line (dN>dS) are under diversifying selection and below (dN<dS) are under purifying selection. The intensity of the point color is proportional to the posterior probability to observe that codon under the selection regimen, calculated with Fubar method.
(TIF)

S3 Fig Selection regimens acting on codons of West Nile Virus polyprotein via MEME method. Using 95 WNV sequences, A) diversifying selection (dN>dS) and B) purifying selection (dN<dS) were estimated. The intensity of the point color is proportional to the posterior probability to observe that codon under the selection regimen, calculated with MEME method. Significant positively selected sites detected by other methods were also included in A).
(TIF)

S1 Table Raw data for FUBAR analysis. (DOCX)

S2 Table Raw data for MEME analysis. (CSV)

S3 Table List of primers used for sequencing. The NS5, envelope and NS5-partial 3’UTR regions were first amplified using flavivirus consensus or West Nile specific primers. This was followed by amplification of NS3 region using designed WNV primers. Finally, specific primers were designed according to the first sequences obtained and a second step of RT-PCR was done to obtain the complete genome.
(DOC)

S1 Dataset Raw growth kinetics data. Raw data for Fig 3.
(XLSX)

S2 Dataset Raw mice survival data. Raw data for Fig 5.
(XLSX)

## References

[1] Kuno G, Chang GJ, Tsuchiya KR, Karabatsos N, Cropp CB. Phylogeny of the genus Flavivirus. J Virol. 1998;72: 73–83. Available: http://jvi.asm.org/content/72/1/73.short 9420202 10.1128/jvi.72.1.73-83.1998 PMC109351

[2] Chambers TJ, Hahn CS, Galler R, Rice CM. Flavivirus genome organization, expression, and replication. Annu Rev Microbiol. 1990;44: 649–88. doi: 10.1146/annurev.mi.44.100190.003245 2174669 10.1146/annurev.mi.44.100190.003245

[3] Gould EA, Zanotto PM de A, Holmes EC. The genetic evolution of flaviviruses. 1997.

[4] Fall G, Diallo M, Loucoubar C, Faye O, Sall AA. Vector competence of Culex neavei and Culex quinquefasciatus (Diptera: Culicidae) from Senegal for lineages 1, 2, Koutango and a putative new lineage of West Nile virus. Am J Trop Med Hyg. 2014;90: 747–754. doi: 10.4269/ajtmh.13-0405 24567319 10.4269/ajtmh.13-0405 PMC3973524

[5] Murgue B, Zeller H, Deubel V. The Ecology and Epidemiology of West Nile Virus in Africa, Europe and Asia In: Mackenzie JS, Barrett ADT, Deubel V, editors. Berlin, Heidelberg: Springer Berlin Heidelberg; 2002 pp. 195–221. doi: 10.1007/978-3-642-59403-8_10 10.1007/978-3-642-59403-8_10 12082990

[6] Hayes EB, Sejvar JJ, Zaki SR, Lanciotti RS, Bode A V., Campbell GL. Virology, pathology, and clinical manifestations of West Nile virus disease. Emerg Infect Dis. 2005;11: 1174–1179. doi: 10.3201/eid1108.050289b 16102303 10.3201/eid1108.050289b PMC3320472

[7] Kramer LD, Li J, Shi P-Y. West Nile virus. Lancet Neurol. 2007;6: 171–181. doi: 10.1016/S1474-4422(07)70030-3 17239804 10.1016/S1474-4422(07)70030-3

[8] Marra PP, Griffing SM, McLean RG. West Nile Virus and Wildlife. Emerg Infect Dis. 2003;9: 7–9. 10.3201/eid0907.030277 PMC3023427 12899147

[9] McLean RG. West Nile Virus: emerging threat to public health and animal health. J Vet Med Educ. 2003;30: 143–4. Available: http://www.ncbi.nlm.nih.gov/pubmed/12970859 12970859 10.3138/jvme.30.2.143

[10] Smithburn KC, Hughes TP, Burke a W, Paul JH, African A. A neurotropic virus isolated from the blood of a native of uganda. Am J Trop Med Hyg. 1940;s1–20: 471–492.

[11] Murgue B, Murri S, Triki H, Deubel V, Zeller HG. West Nile in the Mediterranean basin: 1950–2000. Ann N Y Acad Sci. 2001;951: 117–26. doi: 10.1111/j.1749-6632.2001.tb02690.x 11797769 10.1111/j.1749-6632.2001.tb02690.x

[12] Murray KO, Mertens E, Despres P. West Nile virus and its emergence in the United States of America. Vet Res. 2010;41: 67 doi: 10.1051/vetres/2010039 21188801 10.1051/vetres/2010039 PMC2913730

[13] Hubálek Z, Halouzka J. West Nile fever—a reemerging mosquito-borne viral disease in Europe. Emerg Infect Dis. 1999;5: 643–650. doi: 10.3201/eid0505.990505 10511520 10.3201/eid0505.990505 PMC2627720

[14] ArboNET Arboviral Diseases Branch. Final Maps and Data for 1999–2015. In: Centers for Disease Control and Prevention.

[15] Asnis DS, Conetta R, Waldman G, Teixera AA. The West Nile Virus Encephalitis Outbreak in the United States (1999–2000). Ann N Y Acad Sci. Blackwell Publishing Ltd; 2006;951: 161–171. doi: 10.1111/j.1749-6632.2001.tb02694.x 10.1111/j.1749-6632.2001.tb02694.x 11797774

[16] Nash D, Mostashari F, Fine A, Miller J, O’Leary D, Murray K, et al The Outbreak of West Nile Virus Infection in the New York City Area in 1999. N Engl J Med. 2001;344: 1807–1814. doi: 10.1056/NEJM200106143442401 11407341 10.1056/NEJM200106143442401

[17] Rossi SL, Ross TM, Evans JD. West Nile Virus. Clin Lab Med. 2010;30: 47–65. doi: 10.1016/j.cll.2009.10.006 20513541 10.1016/j.cll.2009.10.006 PMC2905782

[18] Tsai T, Popovici F, Cernescu C, Campbell G, Nedelcu N. West Nile encephalitis epidemic in southeastern Romania. Lancet. 1998;352: 767–771. doi: 10.1016/S0140-6736(98)03538-7 9737281 10.1016/s0140-6736(98)03538-7

[19] Platonov AE, Shipulin GA, Shipulina OY, Tyutyunnik EN, Frolochkina TI, Lanciotti RS, et al Outbreak of West Nile virus infection, Volgograd Region, Russia, 1999. Emerg Infect Dis. 2001;7: 128–32. doi: 10.3201/eid0701.700128 11266303 10.3201/eid0701.010118 PMC2631674

[20] Sambri V, Capobianchi M, Charrel R, Fyodorova M, Gaibani P, Gould E, et al West Nile virus in Europe: emergence, epidemiology, diagnosis, treatment, and prevention. Clin Microbiol Infect. 2013;19: 699–704. doi: 10.1111/1469-0691.12211 23594175 10.1111/1469-0691.12211

[21] Papa A, Bakonyi TT, Xanthopoulou K, Vázquez A, Tenorio A, Nowotny N, et al Genetic Characterization of West Nile Virus Lineage 2, Greece, 2010. Emerg Infect Dis. 2011;17: 920–922. doi: 10.3201/eid1705.101759 21529413 10.3201/eid1705.101759 PMC3321789

[22] Rossini G, Carletti F, Bordi L, Cavrini F, Gaibani P, Landini MP, et al Phylogenetic analysis of west nile virus isolates, Italy, 2008–2009. Emerg Infect Dis. 2011;17: 903–906. doi: 10.3201/eid1705.101569 21529408 10.3201/eid1705.101569 PMC3321781

[23] Bagnarelli P, Marinelli K, Trotta D, Monachetti A, Tavio M, Gobbo R Del, et al Human case of autochthonous West Nile virus lineage 2 infection in Italy, September 2011. Eurosurveillance. 2011;16: 1–4. 22085600

[24] Barzon L, Papa A, Lavezzo E, Franchin E, Pacenti M, Sinigaglia A, et al Phylogenetic characterization of Central/Southern European lineage 2 West Nile virus: analysis of human outbreaks in Italy and Greece, 2013–2014. Clin Microbiol Infect. Elsevier Ltd; 2015;21: 1122.e1–1122.e10. doi: 10.1016/j.cmi.2015.07.018 26235197 10.1016/j.cmi.2015.07.018

[25] Bakonyi T, Ferenczi E, Erdélyi K, Kutasi O, Csörgő T, Seidel B, et al Explosive spread of a neuroinvasive lineage 2 West Nile virus in Central Europe, 2008/2009. Vet Microbiol. 2013;165: 61–70. doi: 10.1016/j.vetmic.2013.03.005 23570864 10.1016/j.vetmic.2013.03.005

[26] Popović N, Milošević B, Urošević A, Poluga J, Lavadinović L, Nedelijković J, et al Outbreak of West Nile virus infection among humans in Serbia, August to October 2012. Euro Surveill. 2013;18: 20613 Available: http://www.eurosurveillance.org/images/dynamic/EE/V18N43/art20613.pdf 24176618 10.2807/1560-7917.es2013.18.43.20613

[27] Pachler K, Lebl K, Berer D, Rudolf I, Hubalek Z, Nowotny N, et al Putative New West Nile Lineage in Uranotaenia unguiculata Mosquitoes, Austria, 2013. Emerg Infect Dis. 2014;20: 2119–2122. doi: 10.3201/eid2012.140921 25418009 10.3201/eid2012.140921 PMC4257835

[28] MacKenzie JS, Williams DT. The zoonotic flaviviruses of southern, south-eastern and eastern Asia, and australasia: The potential for emergent viruses. Zoonoses Public Health. 2009;56: 338–356. doi: 10.1111/j.1863-2378.2008.01208.x 19486319 10.1111/j.1863-2378.2008.01208.x

[29] David S, Abraham AM. Epidemiological and clinical aspects on West Nile virus, a globally emerging pathogen. Infect Dis (Auckl). 2016;48: 571–586. doi: 10.3109/23744235.2016.1164890 27207312 10.3109/23744235.2016.1164890

[30] Rizzoli A, Jiménez-Clavero M, Barzon L, Cordioli P, Figuerola J, Koraka P, et al The challenge of West Nile virus in Europe: knowledge gaps and research priorities. Eurosurveillance. 2015;20: 21135 doi: 10.2807/1560-7917.ES2015.20.20.21135 26027485 10.2807/1560-7917.es2015.20.20.21135

[31] Hall R a, Scherret JH, Mackenzie JS. Kunjin virus: an Australian variant of West Nile? Ann N Y Acad Sci. 2001;951: 153–60. doi: 10.1111/j.1749-6632.2001.tb02693.x 11797773

[32] Lanciotti RS, Roehrig JT, Deubel V, Smith RJ, Parkes M, Steele K, et al Origin of the West Nile Virus Responsible for an Outbreak of Encephalitis in the Northeastern United States. Science (80-). 1999;286: 2333–2337. doi: 10.1126/science.286.5448.2333 10.1126/science.286.5448.2333 10600742

[33] Bakonyi TT, Ivanics E, Erdleyi K, Ursu K, Ferenczi EE, Weissenbock H, et al Lineage 1 and 2 strains of encephalitic West Nile virus, Central Europe. Emerg Infect Dis. 2006;12: 618–623. doi: 10.3201/eid1204.051379 16704810 10.3201/eid1204.051379 PMC3294705

[34] Botha EM, Markotter W, Wolfaardt M, Paweska JT, Swanepoel R, Palacios G, et al Genetic determinants of virulence in pathogenic lineage 2 West Nile virus strains. Emerg Infect Dis. 2008;14: 222–230. doi: 10.3201/eid1402.070457 18258114 10.3201/eid1402.070457 PMC2600181

[35] Beasley DWC, Li L, Suderman MT, Barrett ADT. Mouse Neuroinvasive Phenotype of West Nile Virus Strains Varies Depending upon Virus Genotype. Virology. Elsevier; 2002;296: 17–23. doi: 10.1006/viro.2002.1372 12036314 10.1006/viro.2002.1372

[36] Hubalek Z, Halouzka J, Juricova Z, Sebesta O. First isolation of mosquito-borne west nile virus in the Czech Republic [1]. Acta Virol. 1998;42: 119–120. 9770080

[37] Bakonyi T, Hubálek Z, Rudolf I, Nowotny N. Novel Flavivirus or New Lineage of West Nile Virus, Central Europe. Emerg Infect Dis. 2005;11: 225–231. doi: 10.3201/eid1102.041028 15752439 10.3201/eid1102.041028 PMC3320449

[38] Hubálek Z, Rudolf I, Bakonyi T, Kazdová K, Halouzka J, Šebesta O, et al Mosquito (Diptera: Culicidae) Surveillance for Arboviruses in an Area Endemic for West Nile (Lineage Rabensburg) and Ťahyňa Viruses in Central Europe. J Med Entomol. 2010;47: 466–472. doi: 10.1093/jmedent/47.3.466 20496595 10.1603/me09219

[39] Lvov DK, Butenko AM, Gromashevsky VL, Kovtunov AI, Prilipov AG, Kinney R, et al West Nile virus and other zoonotic viruses in Russia: examples of emerging-reemerging situations In: Calisher CH, Griffin DE, editors. Emergence and Control of Zoonotic Viral Encephalitides. Vienna: Springer Vienna; 2004 pp. 85–96. doi: 10.1007/978-3-7091-0572-6_7 10.1007/978-3-7091-0572-6_7 15119764

[40] Lanciotti RS, Ebel GD, Deubel V, Kerst AJ, Murri S, Meyer R, et al Complete Genome Sequences and Phylogenetic Analysis of West Nile Virus Strains Isolated from the United States, Europe, and the Middle East. Virology. 2002;298: 96–105. doi: 10.1006/viro.2002.1449 12093177 10.1006/viro.2002.1449

[41] Vázquez A, Sánchez-Seco MP, Ruiz S, Molero F, Hernández L, Moreno J, et al Putative new lineage of West Nile virus, Spain. Emerg Infect Dis. 2010;16: 549–552. doi: 10.3201/eid1603.091033 20202444 10.3201/eid1603.091033 PMC3322021

[42] Charrel RN, Brault AC, Gallian P, Lemasson JJ, Murgue B, Murri S, et al Evolutionary relationship between Old World West Nile virus strains: Evidence for viral gene flow between Africa, the Middle East, and Europe. Virology. 2003;315: 381–388. doi: 10.1016/S0042-6822(03)00536-1 14585341 10.1016/s0042-6822(03)00536-1

[43] Prow N a, Setoh YX, Biron RM, Sester DP, Kim KS, Hobson-Peters J, et al The West Nile-like flavivirus Koutango is highly virulent in mice due to delayed viral clearance and the induction of a poor neutralizing antibody response. J Virol. 2014;88: 9947–9962. doi: 10.1128/JVI.01304-14 24942584 10.1128/JVI.01304-14 PMC4136322

[44] Pérez-Ramírez E, Llorente F, del Amo J, Fall G, Lubisi A, Lecollinet S, et al Pathogenicity evaluation of twelve West Nile virus strains belonging to four lineages from five continents in a mouse model: discrimination between three pathogenicity categories. J Gen Virol. Microbiology Society; 2017;98: 662–670. doi: 10.1099/jgv.0.000743 28475031 10.1099/jgv.0.000743

[45] Shope RE. Epidemiology of Other Arthropod-Borne Flaviviruses Infecting Humans. Adv Virus Res. 2003;61: 373–391. doi: 10.1016/S0065-3527(03)61009-2 14714437 10.1016/s0065-3527(03)61009-2

[46] Venter M, Human S, Van Niekerk S, Williams J, van Eeden C, Freeman F. Fatal neurologic disease and abortion in mare infected with lineage 1 West Nile virus, South Africa. Emerg Infect Dis. 2011;17: 1534–1536. doi: 10.3201/eid1708.101794 21801644 10.3201/eid1708.101794 PMC3381566

[47] Virus d’Afrique (base de donnees) [Internet]. [cited 5 Oct 2016]. Available: http://www.pasteur.fr/recherche/banques/CRORA/

[48] Solomon T. West Nile encephalitis. BMJ. 2003;326: 865–869. doi: 10.1136/bmj.326.7394.865 12702624 10.1136/bmj.326.7394.865 PMC1125772

[49] Depoortere E, Kavle J, Keus K, Zeller H, Murri S, Legros D. Outbreak of West Nile virus causing severe neurological involvement in children, Nuba Mountains, Sudan, 2002. Trop Med Int Heal. 2004;9: 730–736. doi: 10.1111/j.1365-3156.2004.01253.x 15189465 10.1111/j.1365-3156.2004.01253.x

[50] Hachfi W, Bougmiza I, Bellazreg F, Bahri O, Kaabia N, Bahri F, et al Une deuxième épidémie de méningo-encéphalite à virus West Nile en Tunisie. Médecine Mal Infect. 2010;40: 456–461. http://dx.doi.org/10.1016/j.medmal.2009.12.005 10.1016/j.medmal.2009.12.005 20079988

[51] Mostashari F, Bunning ML, Kitsutani PT, Singer DA, Nash D, Cooper MJ, et al Epidemic West Nile encephalitis, New York, 1999: results of a household-based seroepidemiological survey. Lancet. Elsevier; 2016;358: 261–264. doi: 10.1016/S0140-6736(01)05480-0 10.1016/S0140-6736(01)05480-0 11498211

[52] Wang W, Sarkodie F, Danso K, Addo-Yobo E, Owusu-Ofori S, Allain J-P, et al Seroprevalence of west nile virus in ghana. Viral Immunol. 2009;22: 17–22. doi: 10.1089/vim.2008.0066 19210224 10.1089/vim.2008.0066

[53] Rappole JH, Derrickson SR, Hubálek Z, Hubálek Z. Migratory birds and spread of West Nile virus in the Western Hemisphere. Emerg Infect Dis. 2000;6: 319–328. doi: 10.3201/eid0604.000401 10905964 10.3201/eid0604.000401 PMC2640881

[54] Renaudet J, Jan C, Ridet J, Adam C, Robin Y. [A serological survey of arboviruses in the human population of Senegal]. Bull Soc Pathol Exot Filiales. 1977;71: 131–140. 33772

[55] Traore-Lamizana M, Zeller HG, Mondo M, Hervy J-P, Adam F, Digoutte J-P. Isolations of West Nile and Bagaza Viruses from Mosquitoes (Diptera: Culicidae) in Central Senegal (Ferlo). J Med Entomol. 1994;31: 934 LP–938. Available: http://jme.oxfordjournals.org/content/31/6/934.abstract 7815413 10.1093/jmedent/31.6.934

[56] Traoré-Lamizana M, Fontenille D, Diallo M, Bâ Y, Zeller HG, Mondo M, et al Arbovirus Surveillance from 1990 to 1995 in the Barkedji Area (Ferlo) of Senegal, a Possible Natural Focus of Rift Valley Fever Virus. J Med Entomol. 2001;38: 480 LP–492. Available: http://jme.oxfordjournals.org/content/38/4/480.abstract 11476327 10.1603/0022-2585-38.4.480

[57] Chevalier V, Dupressoir A, Tran A, Diop OM, Gottland C, Diallo M, et al Environmental risk factors of West Nile virus infection of horses in the Senegal River basin. Epidemiol Infect. Cambridge, UK: Cambridge University Press; 2010;138: 1601–1609. doi: 10.1017/S095026881000035X 20175940 10.1017/S095026881000035X

[58] Shirato K, Miyoshi H, Goto A, Ako Y, Ueki T, Kariwa H, et al Viral envelope protein glycosylation is a molecular determinant of the neuroinvasiveness of the New York strain of West Nile virus. J Gen Virol. 2004;85: 3637–45. doi: 10.1099/vir.0.80247-0 15557236 10.1099/vir.0.80247-0

[59] Moudy RM, Zhang B, Shi P-Y, Kramer LD. West Nile virus envelope protein glycosylation is required for efficient viral transmission by Culex vectors. Virology. 2009;387: 222–8. doi: 10.1016/j.virol.2009.01.038 19249803 10.1016/j.virol.2009.01.038 PMC2742948

[60] Hanna SL, Pierson TC, Sanchez MD, Ahmed AA, Murtadha MM, Doms RW. N-linked glycosylation of west nile virus envelope proteins influences particle assembly and infectivity. J Virol. 2005;79: 13262–74. doi: 10.1128/JVI.79.21.13262-13274.2005 16227249 10.1128/JVI.79.21.13262-13274.2005 PMC1262570

[61] Setoh YX, Prow NA, Hobson-Peters J, Lobigs M, Young PR, Khromykh AA, et al Identification of residues in West Nile virus pre-membrane protein that influence viral particle secretion and virulence. J Gen Virol. 2012;93: 1965–75. doi: 10.1099/vir.0.044453-0 22764317 10.1099/vir.0.044453-0

[62] Beasley DWC, Whiteman MC, Zhang S, Huang CY-H, Schneider BS, Smith DR, et al Envelope protein glycosylation status influences mouse neuroinvasion phenotype of genetic lineage 1 West Nile virus strains. J Virol. 2005;79: 8339–47. doi: 10.1128/JVI.79.13.8339-8347.2005 15956579 10.1128/JVI.79.13.8339-8347.2005 PMC1143769

[63] Brault AC, Huang CY, Langevin SA, Kinney RM, Bowen RA, Ramey WN, et al A single positively selected West Nile viral mutation confers increased virogenesis in American crows. Nat Genet. 2007;39: 1162–1166. doi: 10.1038/ng2097 17694056 10.1038/ng2097 PMC2291521

[64] Ambrose RL, Mackenzie JM. A Conserved Peptide in West Nile Virus NS4A Protein Contributes to Proteolytic Processing and Is Essential for Replication. J Virol. 2011;85: 11274–11282. doi: 10.1128/JVI.05864-11 21880777 10.1128/JVI.05864-11 PMC3194937

[65] Van Slyke G a., Ciota AT, Willsey GG, Jaeger J, Shi PY, Kramer LD. Point mutations in the West Nile virus (Flaviviridae; Flavivirus) RNA-dependent RNA polymerase alter viral fitness in a host-dependent manner in vitro and in vivo. Virology. Elsevier Inc.; 2012;427: 18–24. doi: 10.1016/j.virol.2012.01.036 22365326 10.1016/j.virol.2012.01.036 PMC3299857

[66] Liu WJ, Wang XJ, Clark DC, Lobigs M, Hall R a, Khromykh A a. A Single Amino Acid Substitution in the West Nile Virus Nonstructural Protein NS2A Disables Its Ability To Inhibit Alpha / Beta Interferon Induction and Attenuates Virus Virulence in Mice A Single Amino Acid Substitution in the West Nile Virus Nonstructur. J Virol. 2006;80: 2396–2404. doi: 10.1128/JVI.80.5.2396-2404.2006 16474146 10.1128/JVI.80.5.2396-2404.2006 PMC1395377

[67] Wicker JA, Whiteman MC, Beasley DWC, Davis CT, Zhang S, Schneider BS, et al A single amino acid substitution in the central portion of the West Nile virus NS4B protein confers a highly attenuated phenotype in mice. Virology. 2006;349: 245–253. doi: 10.1016/j.virol.2006.03.007 16624366 10.1016/j.virol.2006.03.007

[68] May FJ, Davis CT, Tesh RB, Barrett ADT. Phylogeography of West Nile virus: from the cradle of evolution in Africa to Eurasia, Australia, and the Americas. J Virol. 2011;85: 2964–74. doi: 10.1128/JVI.01963-10 21159871 10.1128/JVI.01963-10 PMC3067944

[69] Ciccozzi M, Peletto S, Cella E, Giovanetti M, Lai A, Gabanelli E, et al Epidemiological history and phylogeography of West Nile virus lineage 2. Infect Genet Evol. 2013;17: 46–50. doi: 10.1016/j.meegid.2013.03.034 23542457 10.1016/j.meegid.2013.03.034

[70] Brinton M a. Replication cycle and molecular biology of the west nile virus. Viruses. 2013;6: 13–53. doi: 10.3390/v6010013 24378320 10.3390/v6010013 PMC3917430

[71] Carpenter JE, Henderson EP, Grose C. Enumeration of an extremely high particle-to-PFU ratio for Varicella-zoster virus. J Virol. 2009;83: 6917–21. doi: 10.1128/JVI.00081-09 19369328 10.1128/JVI.00081-09 PMC2698559

[72] Alfson KJ, Avena LE, Beadles MW, Staples H, Nunneley JW, Ticer A, et al Particle-to-PFU Ratio of Ebola Virus Influences Disease Course and Survival in Cynomolgus Macaques. Dermody TS, editor. J Virol. 2015;89: 6773–6781. doi: 10.1128/JVI.00649-15 25903348 10.1128/JVI.00649-15 PMC4468478

[73] Hussmann KL, Vandergaast R, Zheng K, Hoover LI, Fredericksen BL. Structural proteins of West Nile virus are a major determinant of infectious particle production and fitness in astrocytes. J Gen Virol. 2014;95: 1991–2003. doi: 10.1099/vir.0.065474-0 24920724 10.1099/vir.0.065474-0 PMC4135089

[74] Engel D, Jöst H, Wink M, Börstler J, Bosch S, Garigliany M, et al Reconstruction of the Evolutionary History and Dispersal of Usutu Virus, a Neglected Emerging Arbovirus in Europe and Africa. MBio. 2016;7: 1–12. doi: 10.1128/mBio.01938-15.Editor 10.1128/mBio.01938-15 PMC4742707 26838717

[75] McMullen AR, Albayrak H, May FJ, Davis CT, Beasley DWC, Barrett ADT. Molecular evolution of lineage 2 West Nile virus. J Gen Virol. 2013;94: 318–25. doi: 10.1099/vir.0.046888-0 23136360 10.1099/vir.0.046888-0 PMC3709619

[76] Hanada K, Suzuki Y, Gojobori T. A large variation in the rates of synonymous substitution for RNA viruses and its relationship to a diversity of viral infection and transmission modes. Mol Biol Evol. SMBE; 2004;21: 1074–1080. doi: 10.1093/molbev/msh109 15014142 10.1093/molbev/msh109 PMC7107514

[77] Berthet FX, Zeller HG, Drouet MT, Rauzier J, Digoutte JP, Deubel V. Extensive nucleotide changes and delections within the envelope glycoprotein gene of Euro-African West Nile viruses. JGenVirol. 1997;78: 2293–2297. Available: http://www.ncbi.nlm.nih.gov/pubmed/9292017 10.1099/0022-1317-78-9-2293 9292017

[78] Butenko AM, Semashko I V, Skvortsova TM, Gromashevskiĭ VL, Kondrashina NG. Detection of the Koutango virus (Flavivirus, Togaviridae) in Somalia. Med Parazitol (Mosk). 1986; 65. 3018465

[79] Jan C, Languillat G, Renaudet J, Robin Y. A serological survey of arboviruses in Gabon. Bull Soc Pathol Exot Filiales. 1978;71: 140 743766

[80] Rizzoli A, Rosà R, Rosso F, Buckley A, Gould E. West Nile Virus Circulation Detected in Northern Italy in Sentinel Chickens. Vector-Borne Zoonotic Dis. 2007;7: 411–417. doi: 10.1089/vbz.2006.0626 17767411 10.1089/vbz.2006.0626

[81] Dow C, Jarrett WFH. Age, strain and sex differences in susceptibility to Cysticercus fasciolaris in the mouse. Exp Parasitol. Elsevier; 1960;10: 72–74. 13724123 10.1016/0014-4894(60)90086-2

[82] Aliota MT, Kramer LD. Replication of West Nile virus, Rabensburg lineage in mammalian cells is restricted by temperature. Parasit Vectors. 2012;5: 293 doi: 10.1186/1756-3305-5-293 23241081 10.1186/1756-3305-5-293 PMC3534007

[83] Moratorio G, Iriarte A, Moreno P, Musto H, Cristina J. A detailed comparative analysis on the overall codon usage patterns in West Nile virus. Infect Genet Evol. 2013;14: 396–400. doi: 10.1016/j.meegid.2013.01.001 23333335 10.1016/j.meegid.2013.01.001

[84] Lo MK, Tilgner M, Bernard K a, Shi P-Y. Functional analysis of mosquito-borne flavivirus conserved sequence elements within 3’ untranslated region of West Nile virus by use of a reporting replicon that differentiates between viral translation and RNA replication. J Virol. 2003;77: 10004–10014. doi: 10.1128/JVI.77.18.10004-10014.2003 12941911 10.1128/JVI.77.18.10004-10014.2003 PMC224605

[85] Brault AC, Kinney RM, Maharaj PD, Green EN, Reisen WK, Huang CY. Replication of the primary dog kidney-53 dengue 2 virus vaccine candidate in Aedes aegypti is modulated by a mutation in the 5’ untranslated region and amino acid substitutions in nonstructural proteins 1 and 3. Vector Borne Zoonotic Dis. 2011;11: 683–689. doi: 10.1089/vbz.2010.0150 21284523 10.1089/vbz.2010.0150

[86] Mandl CW, Holzmann H, Meixner T, Rauscher S, Stadler PF, Allison SL, et al Spontaneous and engineered deletions in the 3’ noncoding region of tick-borne encephalitis virus: construction of highly attenuated mutants of a flavivirus. J Virol. 1998;72: 2132–40. Available: http://www.pubmedcentral.nih.gov/articlerender.fcgi?artid=109508&tool=pmcentrez&rendertype=abstract 9499069 10.1128/jvi.72.3.2132-2140.1998 PMC109508

[87] Brinton MA, Dispoto JH. Sequence and secondary structure analysis of the 5′-terminal region of flavivirus genome RNA. Virology. 1988;162: 290–299. doi: 10.1016/0042-6822(88)90468-0 2829420 10.1016/0042-6822(88)90468-0

[88] Edgar RC. MUSCLE: multiple sequence alignment with high accuracy and high throughput. Nucleic Acids Res. 2004;32: 1792–7. doi: 10.1093/nar/gkh340 15034147 10.1093/nar/gkh340 PMC390337

[89] Rambaut A. Se-Al: sequence alignment editor. 1996;

[90] Strimmer K, von Haeseler a. Quartet puzzling—a quartet maximum-likelihood method for reconstructing tree topologies. Mol Biol Evol. 1996;13: 964–969. doi: 10.1093/oxfordjournals.molbev.a025664

[91] Schmidt HA, Strimmer K, Vingron M, von Haeseler A. TREE-PUZZLE: maximum likelihood phylogenetic analysis using quartets and parallel computing. Bioinformatics. Oxford Univ Press; 2002;18: 502–504. 11934758 10.1093/bioinformatics/18.3.502

[92] Martin D, Rybicki E. RDP: detection of recombination amongst aligned sequences. Bioinformatics. Oxford Univ Press; 2000;16: 562–563. 10980155 10.1093/bioinformatics/16.6.562

[93] Darriba D, Taboada GL, Doallo R, Posada D. jModelTest 2 : more models, new heuristics and parallel computing CircadiOmics : integrating circadian genomics, transcriptomics, proteomics. Nat Methods. Nature Publishing Group; 2012;9: 772 doi: 10.1038/nmeth.2109 22847109 10.1038/nmeth.2109 PMC4594756

[94] Drummond AJ, Suchard MA, Xie D, Rambaut A. Bayesian phylogenetics with BEAUti and the BEAST 1.7. Mol Biol Evol. 2012;29: 1969–73. doi: 10.1093/molbev/mss075 22367748 10.1093/molbev/mss075 PMC3408070

[95] Price MN, Dehal PS, Arkin AP. FastTree 2—approximately maximum-likelihood trees for large alignments. PLoS One. 2010;5: e9490 doi: 10.1371/journal.pone.0009490 20224823 10.1371/journal.pone.0009490 PMC2835736

[96] Stock NK, Laraway H, Faye O, Diallo M, Niedrig M, Sall A a. Biological and phylogenetic characteristics of yellow fever virus lineages from West Africa. J Virol. 2013;87: 2895–907. doi: 10.1128/JVI.01116-12 23269797 10.1128/JVI.01116-12 PMC3571399

[97] Digoutte JP, Calvo-Wilson MA, Mondo M, Traore-Lamizana M, Adam F. Continuous cell lines and immune ascitic fluid pools in arbovirus detection. Res Virol. 1992;143: 417–422. Available: http://www.ncbi.nlm.nih.gov/pubmed/1297177 1297177 10.1016/s0923-2516(06)80135-4

[98] Fall G, Faye M, Weidmann M, Kaiser M, Dupressoir A, Ndiaye EH, et al Real-time RT-PCR assays for detection and genotyping of West Nile virus lineages circulating in Africa. Vector-Borne Zoonotic Dis. Mary Ann Liebert, Inc. 140 Huguenot Street, 3rd Floor New Rochelle, NY 10801 USA; 2016;16: 781–789. doi: 10.1089/vbz.2016.1967 27710313 10.1089/vbz.2016.1967

[99] De Madrid a T, Porterfield JS. A simple micro-culture method for the study of group B arboviruses. Bull World Health Organ. 1969;40: 113–21. Available: http://www.pubmedcentral.nih.gov/articlerender.fcgi?artid=2554446&tool=pmcentrez&rendertype=abstract 4183812 PMC2554446

[100] Wengler G, Wengler G. Cell-associated West Nile flavivirus is covered with E+pre-M protein heterodimers which are destroyed and reorganized by proteolytic cleavage during virus release. J Virol. 1989;63: 2521–2526. 2724410 10.1128/jvi.63.6.2521-2526.1989 PMC250716

[101] Boylan BT, Moreira FR, Carlson TW, Bernard KA. Mosquito cell-derived West Nile virus replicon particles mimic arbovirus inoculum and have reduced spread in mice. PLoS Negl Trop Dis. 2017;11: 1–23. doi: 10.1371/journal.pntd.0005394 28187142 10.1371/journal.pntd.0005394 PMC5322982

[102] Murrell B, Wertheim JO, Moola S, Weighill T, Scheffler K, Kosakovsky Pond SL. Detecting individual sites subject to episodic diversifying selection. PLoS Genet. 2012;8: e1002764 doi: 10.1371/journal.pgen.1002764 22807683 10.1371/journal.pgen.1002764 PMC3395634

[103] Murrell B, Moola S, Mabona A, Weighill T, Sheward D, Kosakovsky Pond SL, et al FUBAR: A Fast, Unconstrained Bayesian Approximation for Inferring Selection. Mol Biol Evol. 2013;30: 1196–1205. doi: 10.1093/molbev/mst030 23420840 10.1093/molbev/mst030 PMC3670733

[104] Pond SLK, Frost SDW, Muse S V. HyPhy: hypothesis testing using phylogenies. Bioinformatics. Springer; 2005;21: 676–679. doi: 10.1093/bioinformatics/bti079 15509596 10.1093/bioinformatics/bti079

[105] Gaunt MW, Sall AA, Lamballerie X De, Gould E, Falconar AKI, Dzhivanian TI, et al Phylogenetic relationships of flaviviruses correlate with their epidemiology, disease association and biogeography. J Gen Virol. 2001;82: 1867–1876. doi: 10.1099/0022-1317-82-8-1867 11457992 10.1099/0022-1317-82-8-1867

[106] Pierre V, Drouet MT, Deubel V. Identification of mosquito-borne flavivirus sequences using universal primers and reverse transcription/polymerase chain reaction. Res Virol. Elsevier; 1994;145: 93–104. 7520190 10.1016/s0923-2516(07)80011-2

