# Supplementary figures and images for: Biological and phylogenetic characteristics of West African lineages of West Nile virus
Source: PLoS Negl Trop Dis. 2017 Nov 8;11(11):e0006078. doi: 10.1371/journal.pntd.0006078 (PMC5695850; doi:10.1371/journal.pntd.0006078)

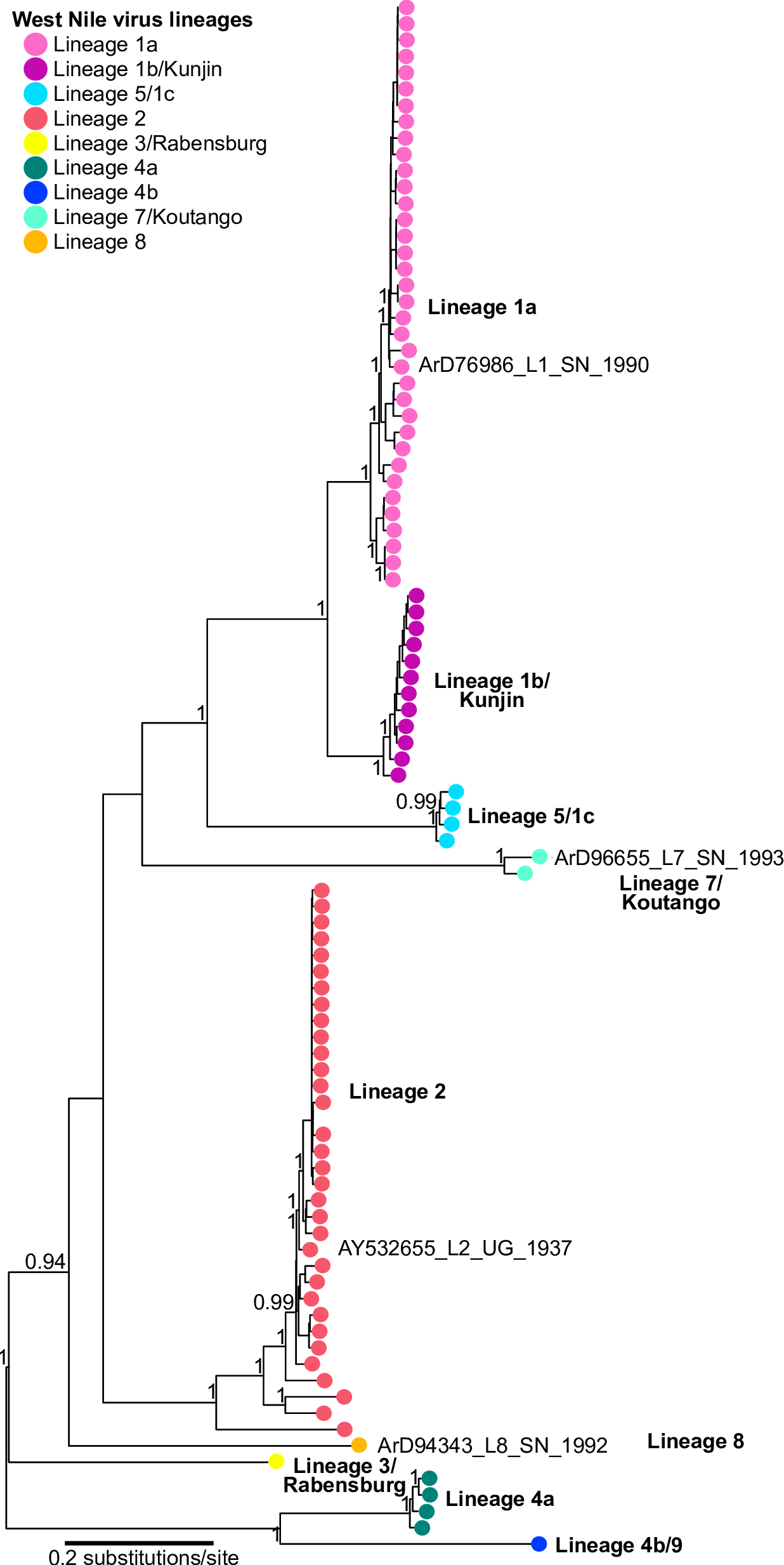

Supplement: S1 Fig — The Shimodaira-Hasegawa values greater than 70% are shown at respective nodes. Tip labels are colored by proposed lineage. Sequences from Table 1 are labeled. (TIF) [file pntd.0006078.s001.tif]

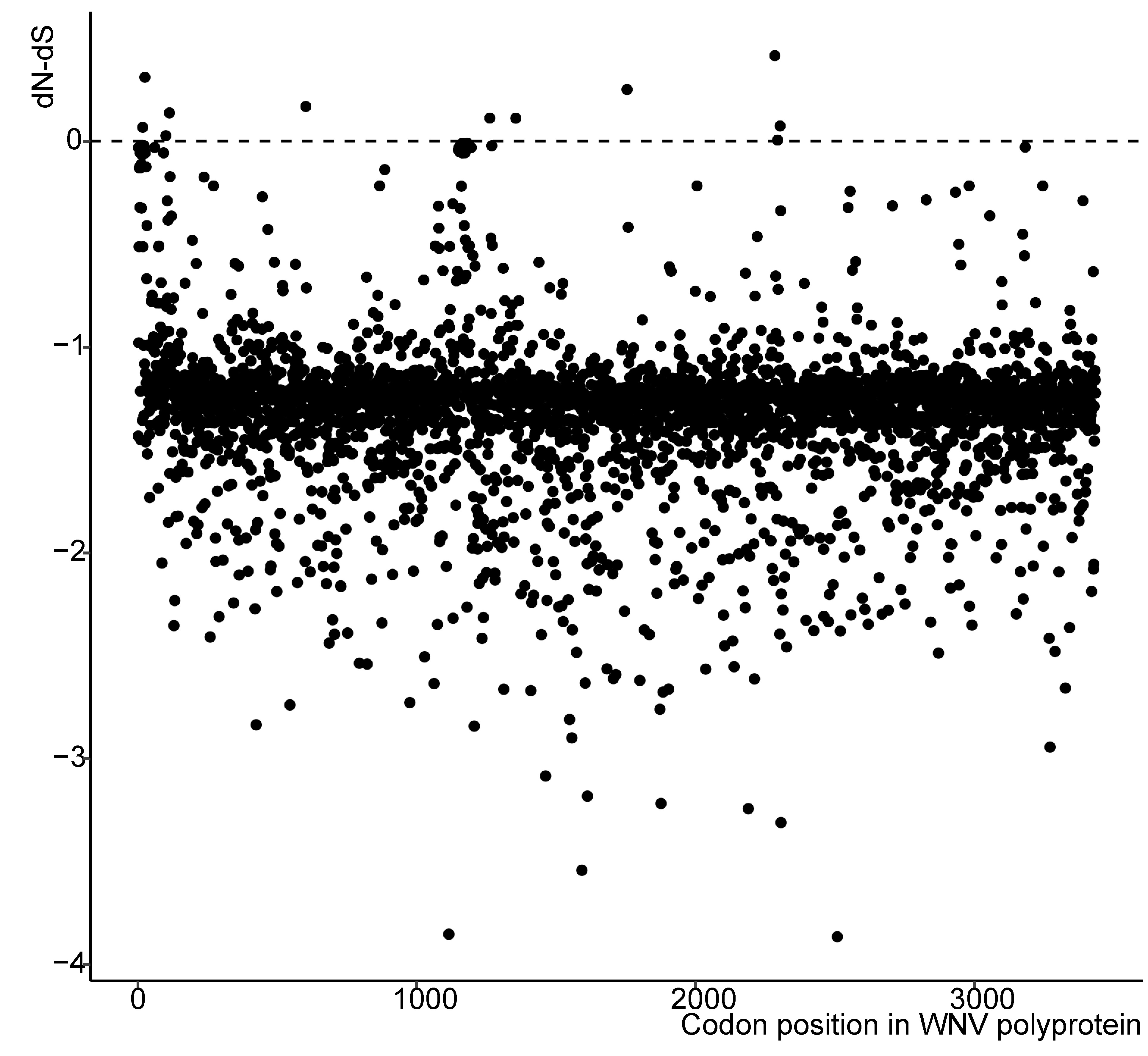

Supplement: S2 Fig — The dashed line marks neutral selection (dN-dS = 0), points above the line (dN>dS) are under diversifying selection and below (dN<dS) are under purifying selection. The intensity of the point color is proportional to the posterior probability to observe that codon under the selection regimen, calculated with Fubar method. (TIF) [file pntd.0006078.s002.tif]

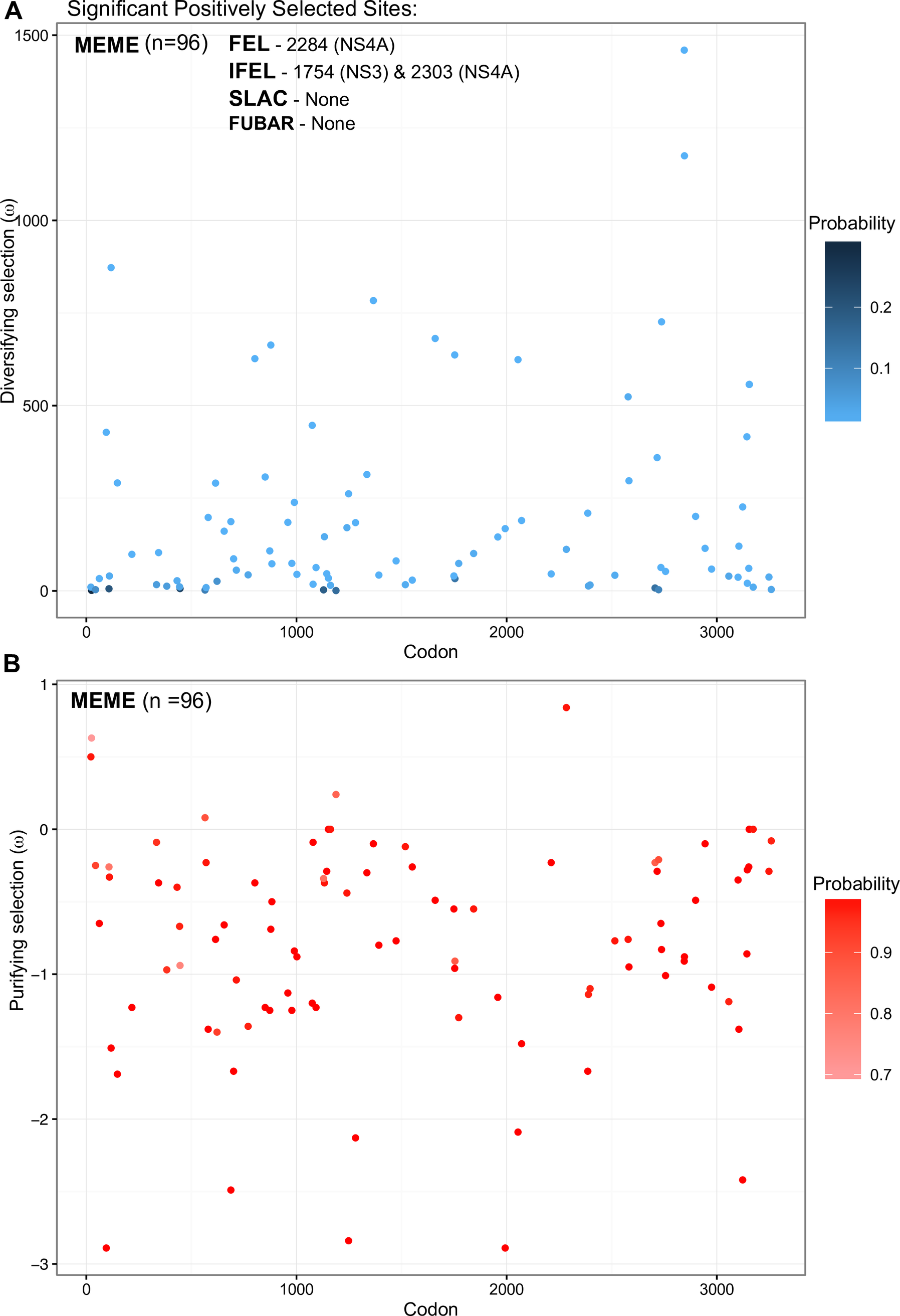

Supplement: S3 Fig — Using 95 WNV sequences, A) diversifying selection (dN>dS) and B) purifying selection (dN<dS) were estimated. The intensity of the point color is proportional to the posterior probability to observe that codon under the selection regimen, calculated with MEME method. Significant positively selected sites detected by other methods were also included in A). (TIF) [file pntd.0006078.s003.tif]
