# Supplementary material for: Biological and phylogenetic characteristics of West African lineages of West Nile virus
Source: PLoS Negl Trop Dis. 2017 Nov 8;11(11):e0006078. doi: 10.1371/journal.pntd.0006078 (PMC5695850; doi:10.1371/journal.pntd.0006078)
Supplement: S3 Table — The NS5, envelope and NS5-partial 3’UTR regions were first amplified using flavivirus consensus or West Nile specific primers. This was followed by amplification of NS3 region using designed WNV primers. Finally, specific primers were designed according to the first sequences obtained and a second step of RT-PCR was done to obtain the complete genome. (DOC) [file pntd.0006078.s006.doc]

| Name | Sequence | Gene | Reference |
| --- | --- | --- | --- |
| FU1PM | TACAACATGATGGGVAARAGWGARAA | NS5 | Kuno et al. 1998 |
| FD3PM | TAC AACATGATGGGVAARAGWGARAA | NS5 | Kuno et al. 1998 |
| E-F | TDGGAATGAGYAACAGRGAC | Envelope | This study |
| E-R | TGRTTDATCTGBTGWTCTCCT | Envelope | This study |
| NS3-F1 | GATGARGTSCARATGATTG | NS3 | This study |
| NS3-R1 | AGTTYTTYCKYTCTTCYCC | NS3 | This study |
| UNIFOR | TGGGGNAAYSRNTGYGGNYTNTTY GG | Envelope | Gaunt et al., 2001 |
| UNIREV | CCNCCHRNNGANCCRAARTCCCA | Envelope | Gaunt et al., 2001 |
| EMF1 | TGGATGACGACGGAAGACATG | NS5 | Pierre et al. 1994 |
| VD8 | GGGTCTCCTCTAACCTCTAGT | 3’UTR | Pierre et al. 1994 |
| WN E-NS3 F | GCTCACAAGAGGGAGCTCTG | Envelope | This study |
| WN E-NS3 R | TTTGATRATCTGTGGCAGDA | NS3 | This study |
| WNK E-NS3 F | GGGACCACTTACGGAGTGTG | Envelope | This study |
| WNK E-NS3 F | GCACATCACATCCACGATCT | NS3 | This study |
| WN NS3-NS5 F | cagggtgcckaactacaatc | NS3 | This study |
| WN NS3-NS5 R | gsgcatcactttcacaactt | NS5 | This study |
| WNK NS3-NS5 F | GGMTTCGAACCTGARATGYT | NS3 | This study |
| WNK NS3-NS5 R | ACGCAGTCATCTCCGCTTAC | NS5 | This study |
| GSP 2-2 R | TDGCRTTBACHGTCATCATC | Capsid | This study |
| WN cap-E F | TCTCTTGGCGTTCTTCAGGT | Capsid | This study |
| WN cap-E R | GGTCAGCACGTTTGTCATTG | Envelope | This study |
| WN NS5- 3UTR F | GACGAACTGGTAGGCAGAGC | NS5 | This study |
| WNK NS5-3UTR F | GATGGAACGTGAAGGAGACC | NS5 | This study |
| WN NS5-3UTR R | CTGGTTGTGCAGAGCAGAAG | 3’UTR | This study |

**Supplementary Table: List of primers used for sequencing**
